# CD52 is a novel target for the treatment of *FLT3*-ITD-mutated myeloid leukemia

**DOI:** 10.1038/s41420-021-00446-8

**Published:** 2021-05-25

**Authors:** Sivasundaram Karnan, Ichiro Hanamura, Akinobu Ota, Souichi Takasugi, Ayano Nakamura, Miyuki Takahashi, Kaori Uchino, Satsuki Murakami, Md Wahiduzzaman, Lam Quang Vu, Md Lutfur Rahman, Muhammad Nazmul Hasan, Toshinori Hyodo, Hiroyuki Konishi, Shinobu Tsuzuki, Kazuhiro Yoshikawa, Susumu Suzuki, Ryuzo Ueda, Masayuki Ejiri, Yoshitaka Hosokawa, Akiyoshi Takami

**Affiliations:** 1 Department of Biochemistry, Aichi Medical University, Nagakute, Aichi Japan; 2 Division of Hematology, Department of Internal Medicine, Aichi Medical University, Nagakute, Aichi Japan; 3 Research Creation Support Center, Aichi Medical University, Nagakute, Aichi Japan; 4 Department of Tumor Immunology, Aichi Medical University, Nagakute, Aichi Japan; 5 Department of Pharmacy, Aichi Medical University, Nagakute, Aichi Japan

**Keywords:** Prognostic markers, Acute myeloid leukaemia

## Abstract

Internal tandem duplication (ITD) of *FMS-like tyrosine kinase 3* (*FLT3*) confers poor prognosis and is found in approximately 25% of cases of acute myeloid leukemia (AML). Although FLT3 inhibitors have shown clinical benefit in patients with AML harboring *FLT3*-ITD, the therapeutic effect is limited. Here, to explore alternative therapeutics, we established a cellular model of monoallelic *FLT3*^ITD/WT^ cells using the CRISPR-Cas9 system in a human myeloid leukemia cell line, K562. cDNA microarray analysis revealed elevated *CD52* expression in K562–FLT3^ITD/WT^ cells compared to K562–FLT3^WT/WT^ cells, an observation that was further confirmed by quantitative real-time-PCR and flow cytometric analyses. The elevated expression of *CD52* in K562–FLT3^ITD/WT^ cells was decreased in wild-type *FLT3* (*FLT3*-WT) knock-in K562–FLT3^ITD/WT^ cells. In K562–FLT3^ITD/WT^ cells, a STAT5 inhibitor, pimozide, downregulated CD52 protein expression while an AKT inhibitor, afuresertib, did not affect CD52 expression. Notably, an anti-CD52 antibody, alemtuzumab, induced significant antibody-dependent cell-mediated cytotoxicity (ADCC) in K562-FLT3^ITD/WT^ cells compared to K562–FLT3^WT/WT^ cells. Furthermore, alemtuzumab significantly suppressed the xenograft tumor growth of K562–FLT3^ITD/WT^ cells in severe combined immunodeficiency (SCID) mice. Taken together, our data suggested that genetically modified *FLT3*-ITD knock-in human myeloid leukemia K562 cells upregulated CD52 expression via activation of STAT5, and alemtuzumab showed an antitumor effect via induction of ADCC in K562–FLT3^ITD/WT^ cells. Our findings may allow establishment of a new therapeutic option, alemtuzumab, to treat leukemia with the *FLT3*-ITD mutation.

## Introduction

The human *FMS-like tyrosine kinase 3* (*FLT3*) gene, which encodes a class III receptor tyrosine kinase, is located on chromosome arm 13q12^1,2^. The gene is expressed in (and displayed on) normal hematopoietic stem cells, and in the acute myeloid leukemia (AML) cells of most patients. The binding of FLT3 to FLT3 ligands activates the intracellular tyrosine kinase domain (TKD) and regulates cell survival, proliferation, and differentiation^1,3,4^. An in-frame *FLT3* internal tandem duplication mutation (*FLT3*-ITD) often occurs in or near the gene sequence encoding the protein’s juxtamembrane (JM) domain^5,6^. *FLT3*-ITD results in ligand-independent dimerization, autophosphorylation, and constitutive activation of downstream signaling pathways, including mitogen-activated protein kinase/extracellular signal-regulated kinase (MAPK/ERK), phosphatidylinositol 3-kinase/AKT, and signal transducer and activator of transcription 5 (STAT5)^7,8^.

*FLT3*-ITD is found in approximately 25% of cases of AML, and 2–4% of cases of chronic myeloid leukemia (CML)^9,10^. Patients with AML having *FLT3*-ITD are highly refractory to conventional chemotherapy^11–13^. Recently developed FLT3 kinase inhibitors are clinically active^14^; however, the treatment outcome of patients with *FLT3*-ITD remains unsatisfactory due to inhibitor insensitivity and/or acquired drug resistance. Therefore, new therapeutic strategies with molecular-level targets, particularly those that differ in their mode of action from classical kinase inhibition, might improve the clinical outcomes of patients with *FLT3*-ITD leukemia.

To this end, detailed investigation and understanding of the molecular mechanisms underlying genetic abnormalities can enable the design of effective targeted therapies. However, limited information is available about *FLT3*-ITD-driven molecular pathologies due to the lack of a proper cellular model; it has been challenging to establish *FLT3*-ITD expression cellular models, other than overexpression models, prior to the development of genome-editing technologies.

Here, we report the successful generation of the first (to our knowledge) cellular model with the *FLT3*-ITD mutation in a human myeloid leukemia cell line, K562, using the CRISPR-Cas9 system. Using this model, we identified a novel *FLT3*-ITD-related gene, *CD52*, through cDNA microarray analysis. Furthermore, we found that alemtuzumab, an anti-CD52 antibody, induced a significant antibody-dependent cell-mediated cytotoxicity (ADCC) in K562–FLT3^ITD/WT^ cells compared to the effect on K562–FLT3^WT/WT^ cells, and dramatically suppressed the growth of xenograft tumors of K562–FLT3^ITD/WT^ cells. Thus, we present the data showing a new potential therapeutic option, alemtuzumab, for the treatment of leukemia with the *FLT3*-ITD mutation.

## Results

### Introduction of *FLT3*-ITD in K562 cells inhibits cell proliferation and colony formation

**Fig. 1:**
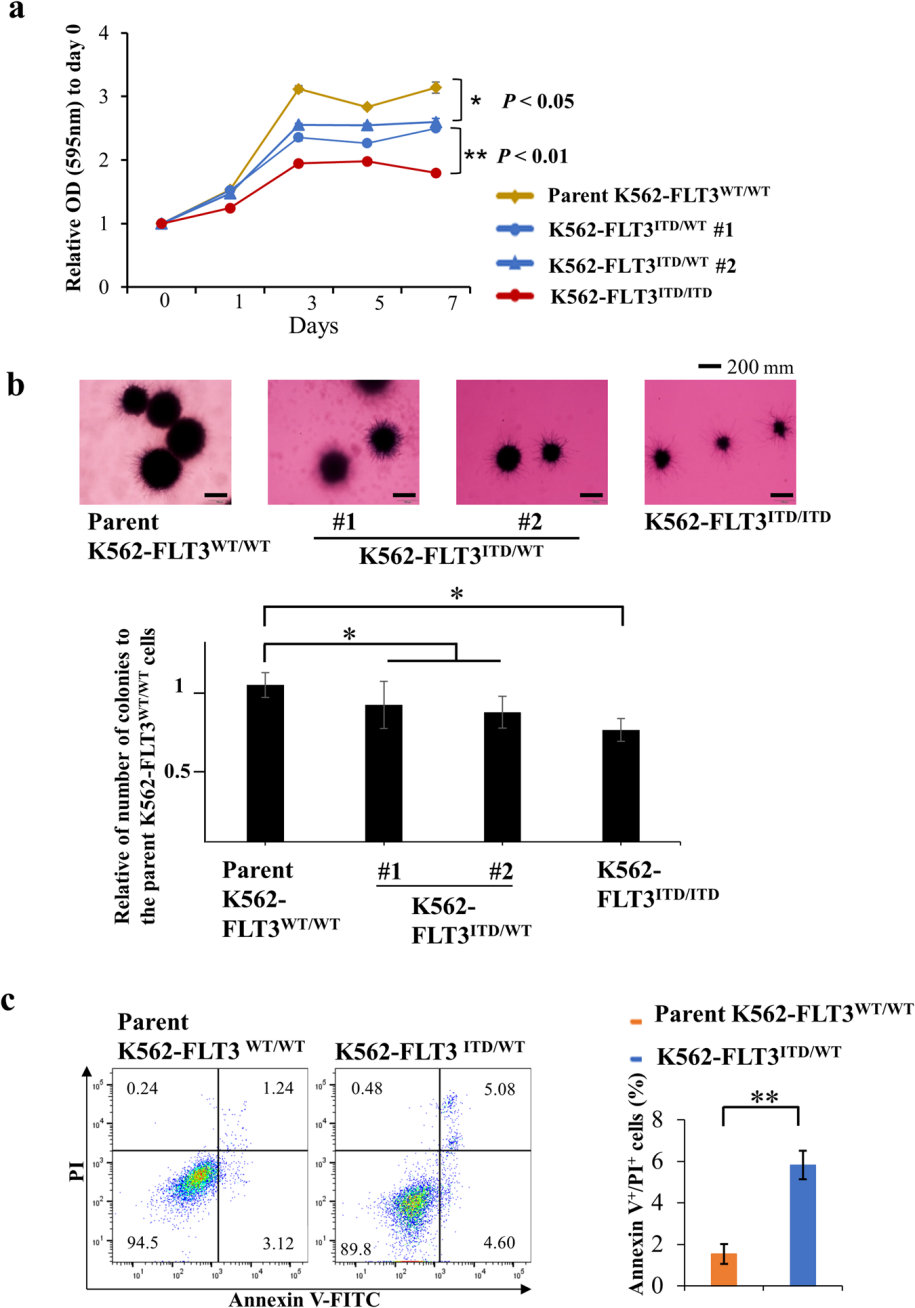
Cell growth and colony formation assay of *FLT3*-ITD knock-in K562 cells. **a** MTT assay for the growth rate of K562 cell clones (yellow, parent K562–FLT3^WT/WT^; blue, K562–FLT3^ITD/WT^; red, K562–FLT3^ITD/ITD^). The relative optical density (OD) at 595 nm on each indicated day was normalized to (divided by) that on Day 0. **b** Representative images of soft agar colony formation assay for the parent K562–FLT3^WT/WT^, K562–FLT3^ITD/WT^, and K562–FLT3^ITD/ITD^ cell clones (upper panel). Number of colonies of indicated K562 cell clones (lower panel). Two hundred cells of each clone were seeded in a six-well plate. After 14 days, the cells were stained with MTT, imaged, and counted. **c** Proportion of apoptotic cells was increased in K562–FLT3^ITD/WT^ cells compared with K562–FLT3^WT/WT^ cells. Data are expressed as mean ± SE (*n* = 3). Asterisks indicate statistically significant differences between indicated K562 cell clones using two-tailed non-paired one-way analysis of variance (ANOVA), followed by post hoc Student’s *t-*test analysis. *p < 0.05, **p < 0.01.

Using the CRISPR-Cas9 system, we established *FLT3*-ITD knock-in K562 cells, including two independent isolates heterozygous for the *FLT3-*ITD allele (K562–FLT3^ITD/WT^ #1 and #2), and one isolate homozygous for the *FLT3*-ITD allele (K562–FLT3^ITD/ITD^) (Supplemental Figs. S1b and S2a–d). We then examined cell proliferation in the parent and mutant strains using an MTT assay. We found that the cell growth was decreased in K562–FLT3^ITD/WT^ cells compared with that in parent K562–FLT3^WT/WT^ cells (*p* < 0.05) (Fig. 1a). The proliferation of K562–FLT3^ITD/ITD^ cells was further decreased compared to that of K562–FLT3^ITD/WT^ cells (*p* < 0.01) (Fig. 1a). In addition, K562–FLT3^ITD/WT^ and K562–FLT3^ITD/ITD^ formed colonies in soft agar that were smaller and decreased in number compared to the parent K562–FLT3^WT/WT^ (*p* < 0.05) (Fig. 1b). We also found that the proportion of apoptotic cells in K562–FLT3^ITD/WT^ was significantly increased compared with that in the parent K562–FLT3^WT/WT^ (*p* < 0.01) (Fig. 1c).

### Gene expression changes induced by the *FLT3*-ITD mutation in K562

**Fig. 2:**
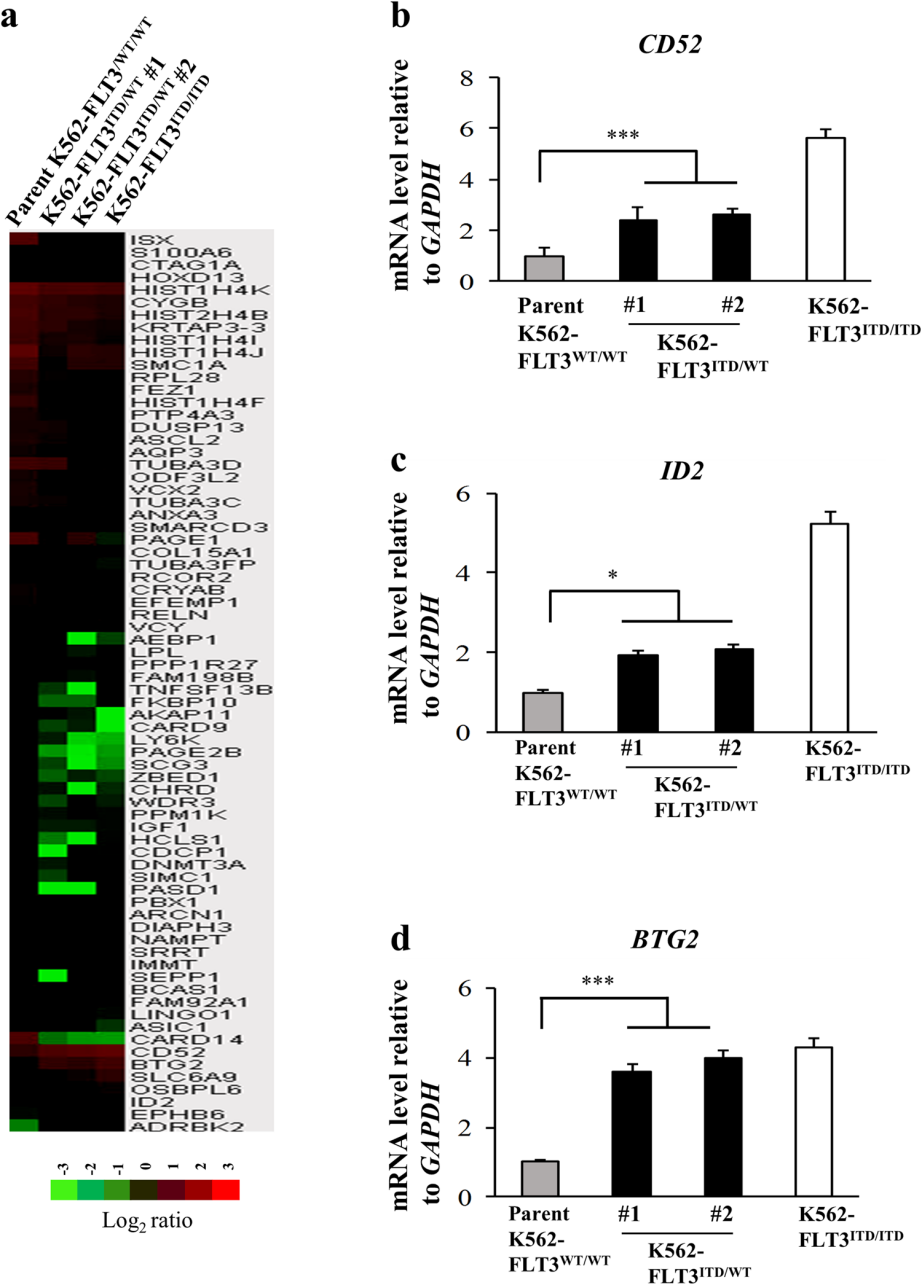
Comparative gene expression profiling and quantitative real-time PCR (qRT-PCR) analysis. **a** A heat map of upregulated or downregulated genes in the parent K562–FLT3^WT/WT^, K562–FLT3^ITD/WT^, and K562–FLT3^ITD/ITD^ cell clones, as determined by microarray analysis. cDNA microarray analysis was performed using the Agilent Whole Human Genome cDNA Microarray Kit (4 × 44 K; Design ID, 026652). A fold change of >2.0 was considered an upregulated gene, and a fold change of <0.5 was considered a downregulated gene. The heat map was constructed with TreeView (Cluster 3.0) software using normalized values for each sample. The corresponding upregulated or downregulated gene names in the heat map are shown on the right side. **b**–**d** Three genes, *CD52*, *BTG2*, and *ID2*, that were upregulated in K562–FLT3^ITD/WT^, and K562–FLT3^ITD/ITD^ cell clones, as determined by the cDNA microarray analysis, were subjected to qRT-PCR analysis in the indicated K562 cell clones using the SYBR Green method. Relative gene expression levels are shown after normalization to *GAPDH* mRNA levels. Data are expressed as mean ± SE (*n* = 3). Asterisks indicate significant differences between parent K562–FLT3^WT/WT^ cells and K562–FLT3^ITD/WT^ using two-tailed non-paired one-way analysis of variance (ANOVA), followed by post hoc Student’s *t-*test analysis. *p < 0.05, ***p < 0.001.

To identify candidate genes related to the *FLT3*-ITD mutation, we performed comprehensive cDNA microarray analysis with parent K562–FLT3^WT/WT^ and K562–FLT3^ITD/WT^ cells. The analysis identified seven genes that were upregulated (>2.0-fold) and 65 genes that were downregulated (<0.5-fold) in K562-FLT3^ITD/WT^ cells compared to the parent K562-FLT3^WT/WT^ cells (Fig. 2a). In addition, clustering of the 72 genes with altered expression showed distinct gene expression patterns in the parent K562-FLT3^WT/WT^ and K562-FLT3^ITD/WT^ cells (Fig. 2a and Supplemental Table S3). We performed qRT-PCR analyses on the candidate genes (identified by microarray analysis above) known to be related to tumorigenesis and/or that encoded cell-surface proteins. We found that the mRNA levels of *CD52*, *BTG2*, and *ID2* were significantly increased, and those of *ISX* and *FEZ1* were significantly decreased, in K562-FLT3^ITD/WT^ cells compared with those in the parent K562-FLT3^WT/WT^ cells (Fig. 2b–d and Supplemental Fig. S3a, b).

### Conversion of the *FLT3*-ITD allele in K562-FLT3^ITD/WT^ cells back to the wild-type sequence decreases expression of *CD52* in the rescued K562–FLT3^WT/WT^ cells

**Fig. 3:**
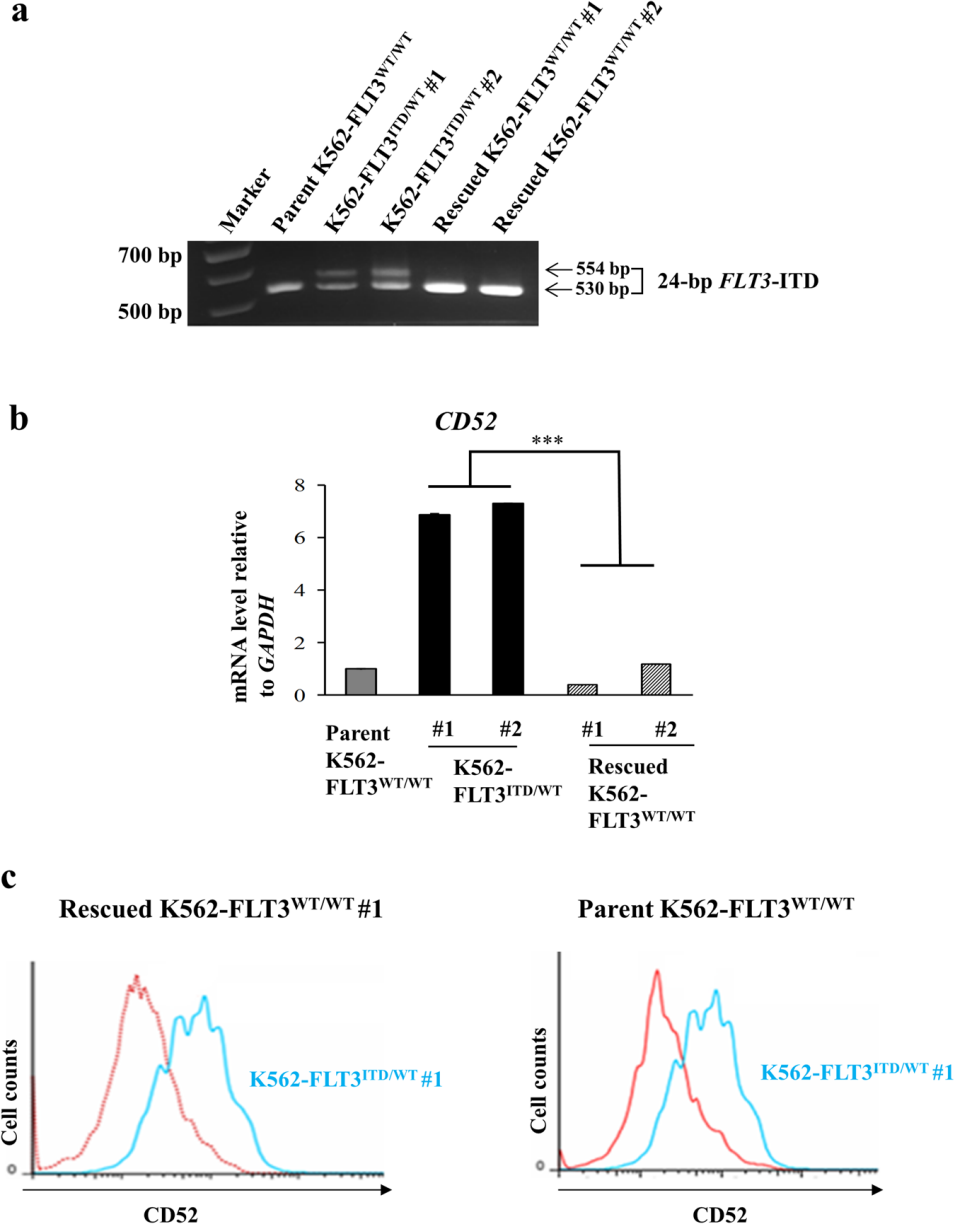
Conversion of *FLT3*-ITD to the wild-type *FLT3* sequence in K562–FLT3^ITD/WT^ cells by the CRISPR-Cas9 system. The *FLT3*-ITD sequence in the K562–FLT3^ITD/WT^ cells was replaced by the wild-type *FLT3* (*FLT3*-WT) sequence. **a** Agarose gel electrophoresis of genomic PCR products containing the *FLT3*-ITD domain in the indicated K562 cells. **b** qRT-PCR analysis of *CD52, BTG2*, and *ID2* genes in the indicated K562 cell clones. Relative gene expression levels are shown after normalization to *GAPDH* mRNA level. Data are expressed as mean ± SE (*n* = 3). Asterisks indicate statistically significant differences between K562–FLT3^ITD/WT^ and rescued K562–FLT3^WT/WT^ using two-tailed non-paired one-way analysis of variance (ANOVA), followed by post hoc Student’s *t-*test analysis. ***p < 0.01. **c** Flow cytometric analysis for CD52 expression in the rescued K562-FLT3^WT/WT^ #1 (red, left panel), the parent K562–FLT3^WT/WT^ (red, right panel), and K562–FLT3^ITD/WT^ #1 (blue, both panels) cells. CD52 expression was decreased in both rescued K562–FLT3^WT/WT^ #1 and parent K562–FLT3^WT/WT^ cells compared with that in K562–FLT3^ITD/WT^ #1 cells.

Since alemtuzumab, a therapeutic antibody against CD52, has been utilized for treatment of chronic lymphocytic leukemia (CLL), we focused on CD52 in subsequent experiments. We converted the *FLT3*-ITD allele (in one heterozygous K562–FLT3^ITD/WT^ isolate) back to the wild-type (WT) sequences using the CRISPR-Cas9 system, yielding a strain that we refer to hereafter as rescued K562–FLT3^WT/WT^ (Fig. 3a), and investigated the expression level of *CD52* in the rescued K562–FLT3^WT/WT^ cells by qRT-PCR. We found that the elevated expression of *CD52* in K562–FLT3^ITD/WT^ cells was significantly decreased in the rescued K562–FLT3^WT/WT^ cells (Fig. 3b). As seen for *CD52* mRNA expression, the elevated cell-surface levels of CD52 protein in K562–FLT3^ITD/WT^ cells were attenuated in the rescued K562-FLT3^WT/WT^ cells, with CD52 protein levels in the rescued strain approaching those seen in parent K562–FLT3^WT/WT^ cells (Fig. 3c). In addition, qRT-PCR analysis also revealed that the elevated expression of *BTG2* and *ID2* in K562–FLT3^ITD/WT^ cells was attenuated in the rescued K562–FLT3^WT/WT^ cells (Supplemental Fig. S4a, b). These results strongly suggested that the expression change of *CD52* observed in the K562–FLT3^ITD/WT^ cells was due to the *FLT3*-ITD mutation.

### Elevated *CD52* expression in patients with AML harboring *FLT3*-ITD

To investigate the relationship between *CD52* expression levels and *FLT3*-ITD in patient samples, we analyzed public domain data (GSE34860) for which *CD52* mRNA expression in patients with AML harboring *FLT3*-ITD is available. We found that the median *CD52* expression in patients with *FLT3*-ITD was nominally (though not significantly) higher than that in patients lacking *FLT3*-ITD (Supplemental Fig. S5). These results suggested that *FLT3*-ITD may increase *CD52* expression in patients with AML.

### *FLT3*-ITD enhances CD52 expression via accumulation of phosphorylated STAT5

**Fig. 4:**
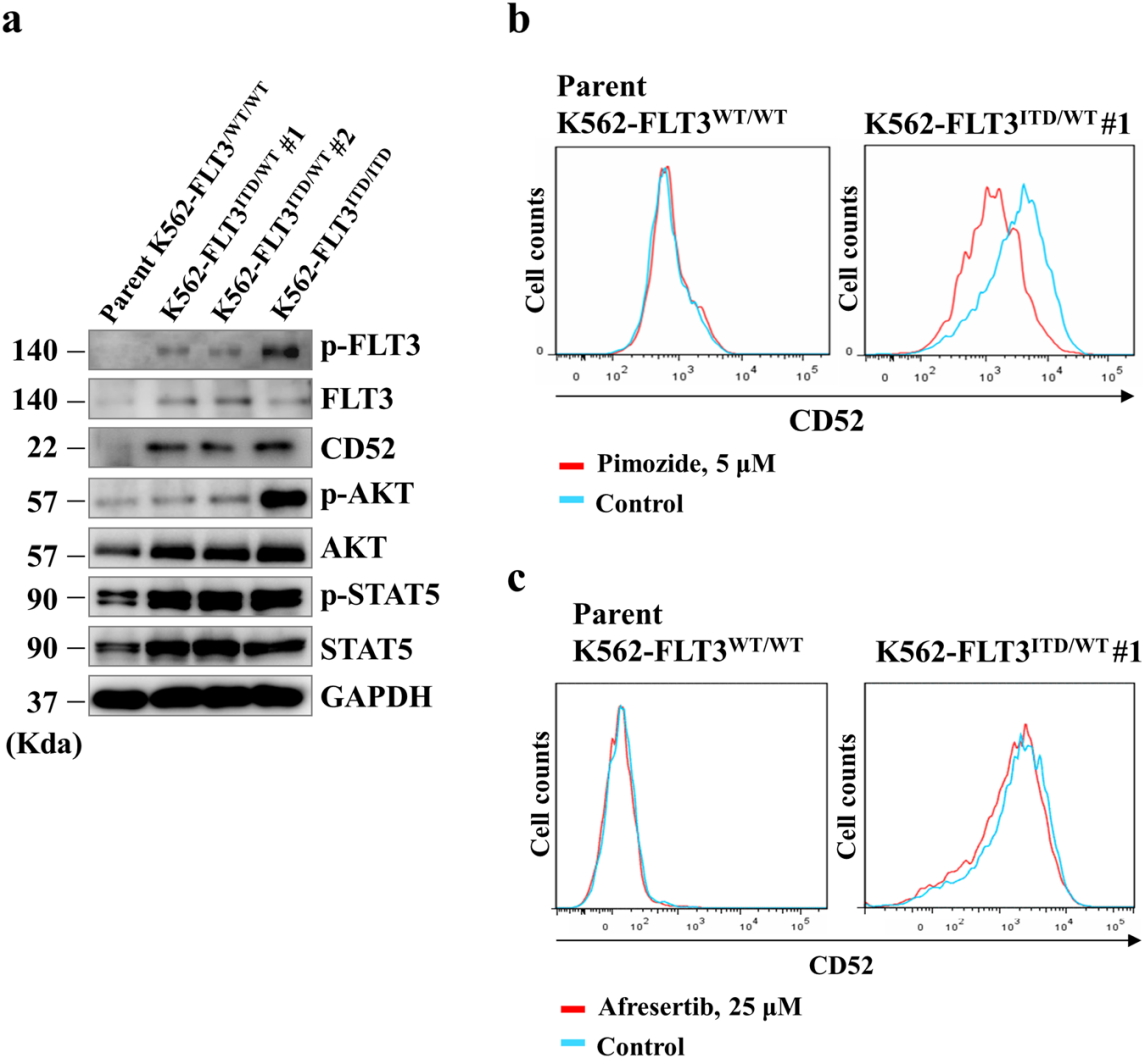
*FLT3*-ITD enhances CD52 expression via upregulation of phosphorylated STAT5. **a** Western blot analysis showing the expression of CD52 and phosphorylation levels of FLT3, AKT, and STAT5 in the indicated clones of K562. **b** Flow cytometric analysis showing the effect of a STAT5 inhibitor, pimozide, on CD52 expression in parent K562–FLT3^WT/WT^ cells (left panel) and K562–FLT3^ITD/WT^ cells (right panel). Cells were treated with 5 μM pimozide for 5 days. **c** Flow cytometric analysis showing the effect of afuresertib, an AKT inhibitor, on CD52 expression in parent K562–FLT3^WT/WT^ cells (left panel) and K562–FLT3^ITD/WT^ cells (right panel). Cells were treated with 25 μM afuresertib for 5 days. Phosphate-buffered saline (PBS) was used as the control.

We next investigated the mechanism of CD52 upregulation in K562–FLT3^ITD/WT^ cells. First, we confirmed the expression of CD52 and the expression and phosphorylation levels of FLT3, AKT, and STAT5 in parent K562–FLT3^WT/WT^, K562–FLT3^ITD/WT^ (K562–FLT3^ITD/WT^ #1 and #2), and K562–FLT3^ITD/ITD^ cells by immunoblotting (Fig. 4a). We observed that, compared to parent K562-FLT3^WT/WT^ cells, the level of phospho-STAT5 was elevated in K562–FLT3^ITD/WT^ cells, and the level of phospho-AKT was further elevated in K562–FLT3^ITD/ITD^ cells (Fig. 4a). Exposure of the cells to pimozide, a STAT5 inhibitor, resulted in decreases in CD52 protein levels in K562–FLT3^ITD/WT^ cells, an effect not seen with afuresertib, an AKT inhibitor (Fig. 4b, c). These results suggested that the accumulation of CD52 protein in cells harboring *FLT3*-ITD occurs via activation of STAT5 transcriptional activity.

### Effects of FLT3 inhibitors on cell proliferation in K562–FLT3^ITD/WT^ cells

**Fig. 5:**
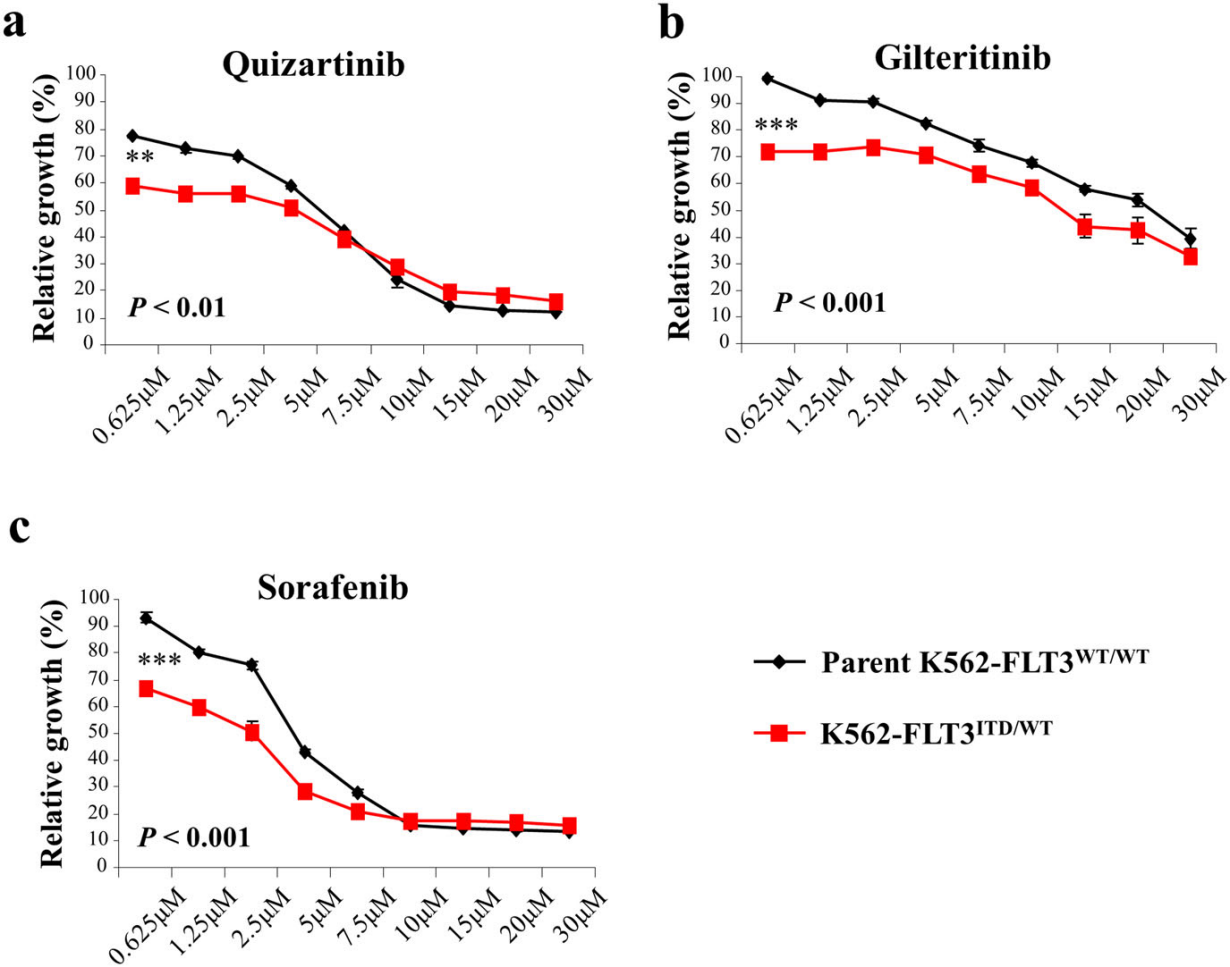
Effect of FLT3 inhibitors on cell proliferation of parent K562–FLT3^WT/WT^ and K562–FLT3^ITD/WT^ cells. **a**–**c** Effect of quizartinib (**a**), gilteritinib (**b**), and sorafenib (**c**) on cell proliferation in parent K562–FLT3^WT/WT^ and K562–FLT3^ITD/WT^ cells. Cells were treated with the indicated concentration of inhibitors for 48 h. Cell growth was measured by the MTT assay. Black and red lines indicate parent K562–FLT3^WT/WT^ cells and K562–FLT3^-ITD/WT^ cells, respectively. Data are expressed as mean ± SE (*n* = 3). Asterisks indicate statistically significant differences between parent K562–FLT3^WT/WT^ cells and K562–FLT3^ITD/WT^ using two-tailed non-paired one-way analysis of variance (ANOVA), followed by post hoc Student’s *t-*test analysis. **p < 0.01, ***p < 0.001.

To clarify the efficacy of FLT3 inhibitors in K562–FLT3^ITD/WT^ cells, we performed MTT assays assessing the growth of K562–FLT3^ITD/WT^ cells in the absence and presence of the indicated FLT3 inhibitors (quizartinib, gilteritinib, and sorafenib). We found that K562–FLT3^ITD/WT^ cells were more sensitive to each of the three FLT3 inhibitors than were parent K562–FLT3^WT/WT^ cells (Fig. 5a–c). These results suggested that FLT3 cellular signaling is functionally active in K562–FLT3^ITD/WT^ cells.

### Comparison of ADCC with alemtuzumab in parent K562–FLT3^WT/WT^ and K562–FLT3^ITD/WT^ cells

**Fig. 6:**
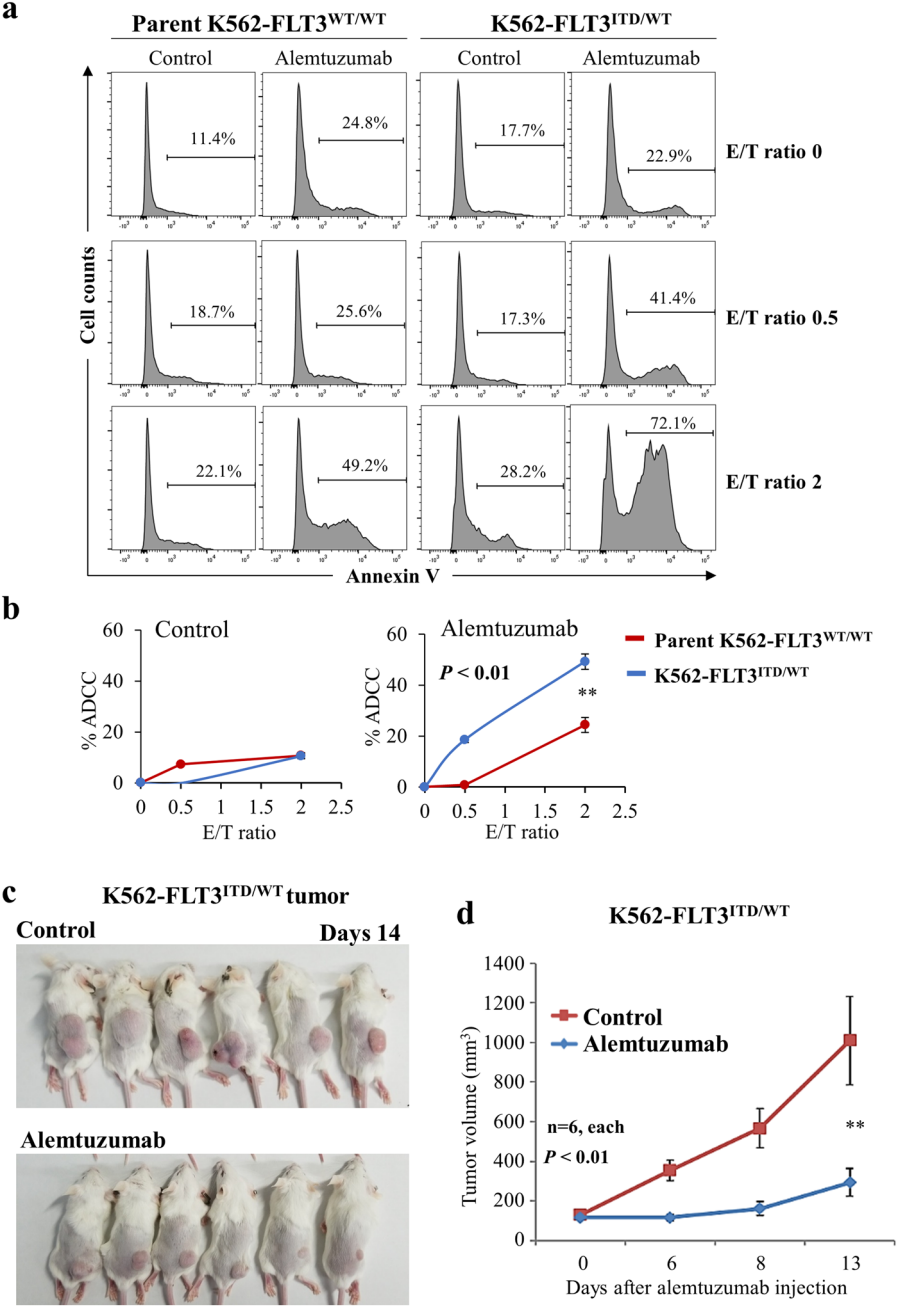
Anti-tumor effects of alemtuzumab in K562–FLT3^ITD/WT^ cells. **a** Comparison of NK cell-mediated antibody-dependent cell-mediated cytotoxicity (ADCC) with alemtuzumab in parent K562–FLT3^WT/WT^ (left panel) and K562–FLT3^ITD/WT^ (right panel) cells at the indicated E/T (effector cell/target cell) ratios. Phosphate-buffered saline (PBS) was used as control for alemtuzumab. **b** Percent ADCC by *E*/*T* ratio with control (left panel) and alemtuzumab (right panel). Red and blue lines indicate parent K562–FLT3^WT/WT^ and K562–FLT3^ITD/WT^ cells, respectively. Alemtuzumab showed higher percent ADCC in K562–FLT3^ITD/WT^ cells than that in parent K562–FLT3^WT/WT^ cells, whereas control did not. Data are expressed as mean ± SE (*n* = 3). **c**, **d** Effect of alemtuzumab on the tumor growth of xenografted K562–FLT3^ITD/WT^ cells. **c** Mice implanted with tumors of xenografted K562–FLT3^ITD/WT^ cells and treated with control (PBS) or alemtuzumab are pictured at the time of euthanasia on day 14. **d** Tumor volume of xenografted K562–FLT3^ITD/WT^ cells treated with control or alemtuzumab on the indicated day. Data are expressed as mean ± SE (*n* = 6). Asterisks indicate statistically significant differences between control and alemtuzumab and K562–FLT3^ITD/WT^ using two-tailed non-paired one-way analysis of variance (ANOVA), followed by post hoc Student’s *t-*test analysis. **p < 0.01.

Alemtuzumab-induced NK cell-mediated ADCC was examined in parent K562–FLT3^WT/WT^ and K562–FLT3^ITD/WT^ cells. ADCC with alemtuzumab was consistently elevated in K562–FLT3^ITD/WT^ cells compared to that in parent K562–FLT3^WT/WT^ cells, regardless of the E/T (effector cell/target cell) ratio (*p* < 0.01) (Fig. 6a, b).

### Alemtuzumab inhibits xenograft tumor growth of K562–FLT3^ITD/WT^ cells in SCID mice

We examined the effect of alemtuzumab on in vivo tumor growth of K562–FLT3^ITD/WT^ cells using a xenograft tumor model in SCID mice. As expected, the growth of K562–FLT3^ITD/WT^ tumors was significantly attenuated in mice treated with alemtuzumab compared to tumor growth in tumor-bearing mice treated with vehicle alone (*p* < 0.01) (Fig. 6c, d).

### Cytotoxic effects of alemtuzumab on AML cells with *FLT3*-ITD

**Fig. 7:**
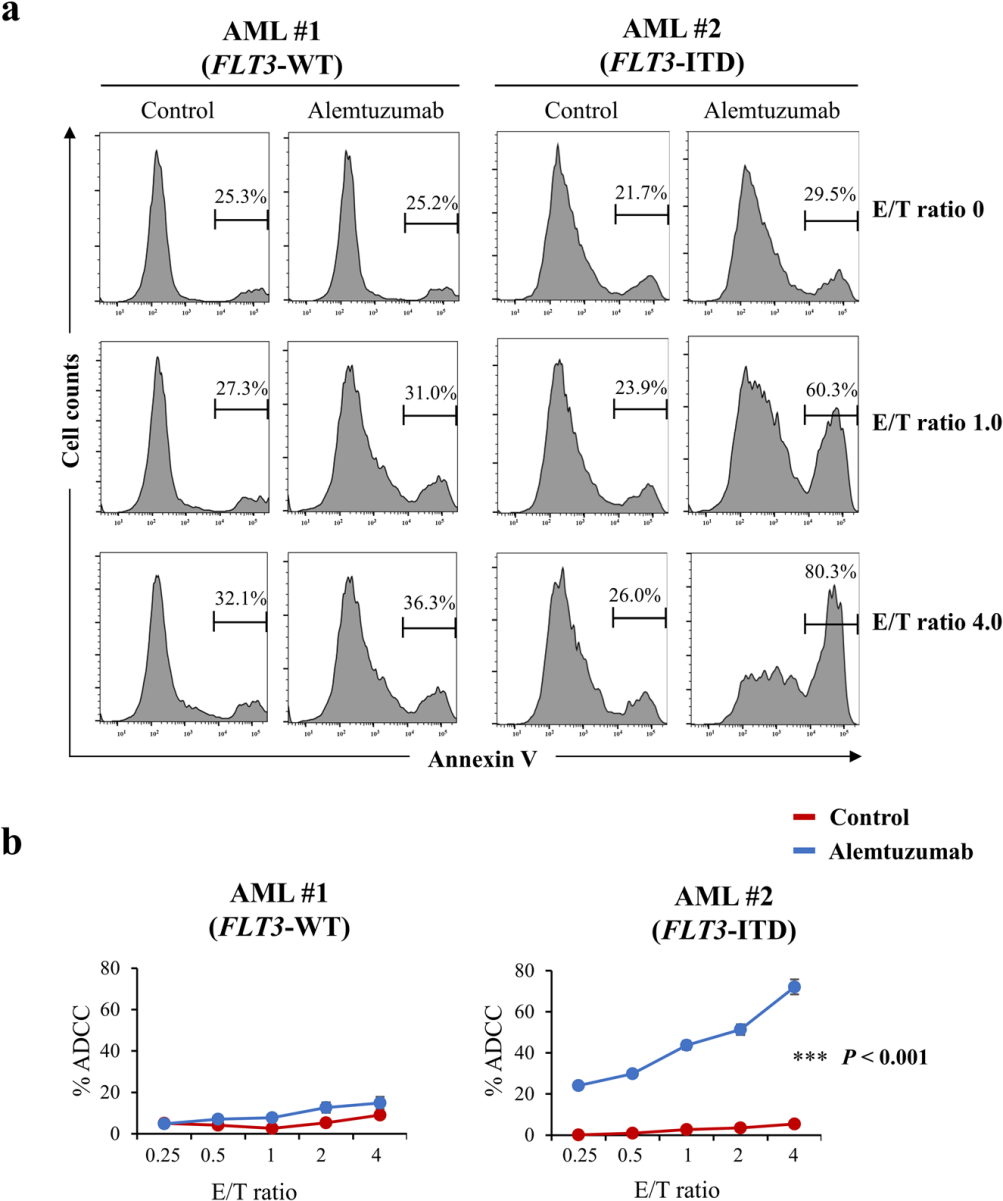
Effects of alemtuzumab in human primary AML cells. **a** Comparison of NK cell-mediated cytotoxicity (antibody-dependent cellular cytotoxicity, ADCC) with alemtuzumab in primary cells from an AML patient with *FLT3*-WT (AML #1, left panel) or from an AML patient with *FLT3*-ITD (AML #2, right panel) cells at the indicated *E*/*T* (effector cell/target cell) ratios. Phosphate-buffered saline (PBS) was used as control for alemtuzumab. **b** Percent ADCC by E/T ratio with control (red line) and alemtuzumab (blue line) in cells from AML patients. Alemtuzumab showed higher percent ADCC in the cells from the AML patient with *FLT3*-ITD (right panel) than that in those from the AML patient with *FLT3*-WT (left panel). Data are expressed as mean ± SE (*n* = 3). Asterisks indicate statistically significant differences between control and alemtuzumab in indicated cells using two-tailed non-paired one-way analysis of variance (ANOVA), followed by post hoc Student’s *t-*test analysis. ***p < 0.001.

Finally, we performed the ADCC assay with alemtuzumab on newly diagnosed AML patient samples that harbor the *FLT3*-ITD mutation. We found that alemtuzumab showed ADCC in cells from an AML patient with *FLT3*-ITD, but did not show ADCC in cells from an AML patient with *FLT3*-WT (Fig. 7a, b). In addition, alemtuzumab showed the ADCC in MOLM-13 cells, a human AML cell line harboring *FLT3*-ITD, which was slightly resistant to quizartinib compared with MOLM-14 cells which were also a human AML cell line with *FLT3*-ITD (Supplementary Fig. S7a–c).

## Discussion

In this study, we generated a cellular model of the *FLT3*-ITD mutation using the CRISPR-Cas9 system and a human myeloid leukemia cell line, K562 (Supplemental Figs. S1a, b and S2a–d). We found that the expression of *CD52* (at both the mRNA and protein level) was increased in *FLT3*-ITD knock-in K562 cells (K562–FLT3^ITD/WT^ cells) compared to the parent and to rescued K562–FLT3^WT/WT^ cells (Figs. 2a, b and 3a–c). Furthermore, our analysis with a database deposited in the public domain showed that *CD52* mRNA expression in samples from patients with *FLT3*-ITD-positive AML tended to be higher than that in samples from patients with *FLT3*-ITD-negative AML (Supplemental Fig. S5). Together, these results indicated the possibility that *FLT3*-ITD cellular signaling is closely associated with *CD52* expression. Moreover, we found that alemtuzumab, an anti-CD52 antibody, induced stronger ADCC in K562–FLT3^ITD/WT^ cells compared with that in K562–FLT3^WT/WT^ cells (Fig. 6a, b) and dramatically suppressed tumor growth by K562–FLT3^ITD/WT^ cells in mouse xenograft experiments (Fig. 6c, d). Additionally, we demonstrated that alemtuzumab showed ADCC in cells from AML patients with *FLT3*-ITD and MOLM-13 cells that had *FLT3*-ITD (Fig. 7a, b and Supplementary Fig. S7a–c). To our knowledge, this work is the first to describe the generation of *FLT3*-ITD mutants using human myeloid leukemia cells and the CRISPR-Cas9 system; these cells lines allowed us to elucidate the relationship between the *FLT3*-ITD mutation and *CD52* overexpression. Our findings suggest the possibility of a new therapeutic option, the anti-CD52 antibody alemtuzumab, to treat leukemia carrying the *FLT3*-ITD mutation.

We demonstrated that *FLT3*-ITD was associated with increased expression of CD52 in genetically modified *FLT3*-ITD knock-in K562 cells and in patients with AML (Fig. 2a, b and Supplemental Fig. S5). Multiple studies have investigated the molecular mechanisms and cell phenotype underlying *FLT3*-ITD; however, we are not aware of any reports examining the relationship between *FLT3*-ITD and CD52. This issue may reflect differences in the methods used to establish transfectants harboring the *FLT3*-ITD mutation. In cells into which *FLT3*-ITD is introduced by a conventional method (e.g., using a viral vector), the ectopic *FLT3*-ITD gene would be overexpressed, potentially resulting in a phenotype distinct from that of actual patient leukemia cells, which typically may not overexpress the mutant gene. In contrast, we employed genome editing, permitting the construction of a *FLT3*-ITD-containing leukemia cell model that more closely resembles the actual condition of leukemia cells in patients. Thus, genome editing may have revealed alterations of cell phenotype specific to *FLT3*-ITD that previously have been overlooked. We also found that *ID2* and *BTG2* were upregulated in K562–FLT3^ITD/WT^ cells compared to parent K562–FLT3^WT/WT^ cells (Fig. 2c, d). The molecular function and significance of the elevated expression of these genes will need to be examined in future studies.

*FLT3*-ITD has been reported to activate PI3K/AKT signaling via STAT5-mediated activation in AML cells^15–17^. In the present work, we confirmed that phospho-STAT5 levels were increased in K562–FLT3^ITD/WT^ cells compared to parental cells; additionally, the levels of phospho-AKT appeared to show progressive increases when comparing K562–FLT3^WT/WT^, K562–FLT3^ITD/WT^, and K562–FLT3^ITD/ITD^ cells (Fig. 4a). Regarding the relationship between cellular signaling and CD52 expression, we observed that CD52 expression in K562–FLT3^ITD/WT^ cells was decreased upon exposure to pimozide, a STAT5 inhibitor, whereas exposure to afuresertib, an AKT inhibitor, had no significant effect of on CD52 expression in K562-FLT3^ITD/WT^ cells (Fig. 4b, c). This observation suggested that signaling by STAT5, but not that by AKT, is involved in *FLT3*-ITD-induced upregulation of CD52.

Using MMT and colony formation assays, we found that cell growth was decreased in the K562–FLT3^ITD/WT^ and K562–FLT3^ITD/ITD^ cells compared with that in the parent K562–FLT3^WT/WT^ cells (Fig. 1a, b). These findings differ from those of the previous literature, which reported that *FLT3*-ITD enhanced cell proliferation^18^. We speculate that the impaired growth seen in the present work reflects oncogenic death^19^ in our *FLT3*-ITD cells caused by excessive strong proliferation signaling resulting from the additional FLT3 cellular signaling in K562 cells, that already are subject to BCR-ABL tyrosine kinase activity.

CD52, which also is known as CAMPATH-1, is a glycoprotein that is expressed on the cell surface of lymphocytes, monocytes, and dendritic cells^20^. Alemtuzumab, a humanized anti-CD52 antibody, has been used for depleting lymphocytic leukemia cells in patients with CLL. Alemtuzumab induces the killing of CD52-positive lymphocytes via ADCC and complement-dependent cytotoxicity^20,21^. In the present study, alemtuzumab exposure provided increased ADCC in K562–FLT3^ITD/WT^ cells compared with that in parent K562–FLT3^WT/WT^ cells (Fig. 6a, b), and dosing with alemtuzumab suppressed the growth of K562–FLT3^ITD/WT^ tumors in a mouse xenograft model (Fig. 6c, d). These results suggested that alemtuzumab suppresses, via ADCC, the in vivo growth of K562–FLT3^ITD/WT^ cells, which have elevated expression of CD52. Interestingly, a previous study reported that the administration of alemtuzumab in NOD SCID gamma (NSG) mice significantly suppressed the engraftment of AML patient-derived CD52-positive leukemia cells with the *FLT3*-ITD mutation, while not suppressing engraftment of leukemia cells lacking the *FLT3*-ITD mutation^22^. These data suggest that alemtuzumab may be effective for the treatment of myeloid leukemia with *FLT3*-ITD. Additionally, alemtuzumab has been employed as a therapeutic option for reducing the risk of graft-versus-host disease (GVHD) by eliminating lymphocytes^23^. Therefore, it may be possible to improve the outcome of cases with *FLT3*-ITD myeloid leukemia using alemtuzumab as a GVHD prophylaxis for patients undergoing allogeneic hematopoietic stem cell transplantation.

In addition to our modification of K562 cells, we established LCL-FLT3-ITD^ITD/WT^ cells. We demonstrated that LCL-FLT3-ITD^ITD/WT^ cells showed increased expression of *CD52* mRNA, decreased cell proliferation, and increased levels of apoptosis compared to the parent LCL-FLT3-ITD^WT/WT^ cells (Supplemental Fig. S6a–d). These results suggest that *FLT3*-ITD effects on *CD52* expression and cell proliferation are not unique to K562 cells.

We found that FLT3 inhibitors (quizartinib, gilteritinib and sorafenib) suppress the proliferation of K562–FLT3^ITD/WT^ cells compared with that of parent K562–FLT3^WT/WT^ cells (Fig. 5a–c), suggesting that FLT3 signaling is functionally active in K562–FLT3^ITD/WT^ cells. Although FLT3 inhibitors can prolong the survival of patients harboring cancers with *FLT3*-ITD^24^, the outcome of treatment in these patients remains unsatisfactory due to insensitivity to these compounds and/or acquired drug resistance^25,26^. Secondary mutations in the sequences encoding the FLT3 TKD have been observed in a subset of those patients^27,28^. Therefore, an anti-tumor antibody targeting CD52, such as alemtuzumab, is a potential therapeutic alternative for AML with *FLT3*-ITD, given that this antibody targets and kills leukemic cells by a mechanism that is different from that of the FLT3 kinase inhibitors.

A major limitation of our study is that we did not analyze many primary samples. Further studies investigating ADCC with alemtuzumab, and the anti-tumor effects of alemtuzumab in a xenograft model using more patient samples will be needed to validate our proposed treatment strategy for *FLT3*-ITD leukemia.

Taken together, the findings of this study revealed CD52 as a molecular target for the antibody treatment of *FLT3*-ITD leukemia. Since alemtuzumab is an approved drug, further clinical studies using alemtuzumab are warranted to evaluate our proposed treatment strategy for *FLT3*-ITD leukemia. The present study offers valuable clues for further improvement of the outcomes in this challenging disease.

## Materials and methods

### Cell culture and reagents

The human CML cell line K562 was obtained from the Japanese Collection of Research Bioresource Cell Bank. The human AML cell line MOLM-13 and MOLM-14 were purchased from DSMZ (German Collection of Microorganisms and Cell Cultures). Primary human AML samples from newly diagnosed patients were obtained from the sample archive at the Aichi Medical University Hospital. All samples were obtained after written informed consent, and the use of biological samples for research was approved by the ethics committee of the Aichi Medical University Hospital (Approval Number 2020-156), in accordance with the Declaration of Helsinki. The cells were cultured in RPMI-1640 supplemented with 10% fetal bovine serum (FBS) at 37 °C in a 5% CO_2_ humidified atmosphere. Afuresertib, gilteritinib, and quizartinib were purchased from Selleck Chemicals (Houston, TX, USA). Sorafenib was obtained from ChemScene (Monmouth Junction, NJ, USA). Pimozide was obtained from Sigma-Aldrich (St. Louis, MO, USA). Alemtuzumab, a recombinant humanized monoclonal antibody against human CD52, was purchased from Sanofi K.K. (Tokyo, Japan).

### Establishment of genetically *FLT3*-ITD knock-in cell clones generated using the CRISPR-Cas9 system

The CRISPR-Cas9 system was used to convert the wild-type sequence of *FLT3* (*FLT3*-WT) to *FLT3*-ITD, as described elsewhere^29^. pSpCas9 (BB)-2A-GFP (PX458) was a gift from Dr Feng Zhang (Addgene plasmid # 48138; Addgene)^30^. A single guide RNA (sgRNA) sequence for *FLT3* was selected using Optimized CRISPR Design (http://crispr.mit.edu/). The sgRNA sequence for *FLT3* exon 14 was 5′-GTAGAAGTACTCATTATCTG-3′ (Supplemental Fig. S1a). A 1034-bp DNA fragment containing the 24-bp *FLT3*-ITD sequence was obtained by PCR amplification of genomic DNA from a human myeloid leukemia cell line, AMU-AML2 cells, previously established in our laboratory. Amplification of the *FLT3*-ITD gene was performed using primers as follows: forward (Fw), 5′-ACTCAAGTGATCCTCCCATC-3′, and reverse (Rev), 5′-TGACTGGGTTGACACCCCA-3′. The DNA fragment containing the 24-bp *FLT3*-ITD sequence was inserted into a plasmid vector, pcDNA 3.1 (+), using TA cloning, yielding a plasmid that was designated pcDNA/*FLT3*-ITD. The sgRNA sequence for *FLT3* was cloned by ligating corresponding oligonucleotides into the BbsI site of PX458, yielding a plasmid designated FLT3/PX458. To convert *FLT3*-WT to *FLT3*-ITD in K562 cells, FLT3/PX458 and pcDNA/*FLT3*-ITD were co-transfected into K562 cells using a 4D-Nucleofector™ instrument (Lonza Japan, Tokyo, Japan). A single clone was selected from a 96-well plate, expanded in a 12-well plate, and then used for biological assays. For sequence analysis and agarose gel electrophoresis analysis, the *FLT3* gene was amplified by PCR using the following primers: Fw, 5′-AGAAGTGGAAGAAGAGGTGG-3′, and Rev, 5′-TCCAAGACAACATCTCATTC-3′. The *FLT3* gene amplified from genomic DNA was subjected to sequence analysis to confirm the monoallelic presence of the 24-bp duplication (*FLT3*-ITD) in two independent K562–FLT3^ITD/WT^ clones (designated #1 and #2), and the biallelic presence of the 24-bp duplication in one K562–FLT3^ITD/ITD^ clone (Supplemental Fig. S1a–d). To convert the *FLT3*-ITD allele in K562–FLT3^ITD/WT^ cells to the *FLT3*-WT sequence, 1 μg each of FLT3/PX458 and pcDNA/*FLT3*-WT were co-transfected into K562–FLT3^ITD/WT^ cells (1 × 10^6^ cells/mL) using a 4D-Nucleofector™ as above.

### Construction of model cell line using lymphoblastoid cell line (LCL) cell line

A human B-cell-derived LCL was kindly provided by Dr. Sonta Shin-ichi (Fetal Life Science Center, Aichi, Japan). We established a LCL cell clone containing the monoallelic 24-bp duplication of the *FLT3*-ITD mutation using the CRISPR-Cas9 system as above (Supplemental Fig. S2a). The resulting clone was designated LCL-FLT3^ITD/WT^.

### cDNA microarray analysis

cDNA microarray analysis was performed according to the manufacturer’s protocol (Agilent Technologies, Santa Clara, CA, USA). Briefly, cDNA synthesis and cRNA labeling with cyanine 3 (Cy3) dye were performed using the Agilent Low Input Quick Amp Labeling Kit (Agilent Technologies). Cy3-labeled cRNA then was purified, fragmented, and hybridized to a Human Gene Expression 4x44K v2 Microarray Chip containing 27,958 Entrez Gene RNAs using a Gene Expression Hybridization Kit (Agilent Technologies). To compare gene expression profiles between the parent K562–FLT3^WT/WT^ cells and K562–FLT3^ITD/WT^ cells, raw fluorescence values were normalized and clustered according to the differential gene expression. The raw and normalized microarray data have been submitted to the GEO database at NCBI as Accession Number GSE116727.

### Quantitative reverse transcription real-time PCR (qRT-PCR)

qRT-PCR analyses for *FLT3*, *CD52* (*cluster of differentiation 52*), *ID2* (*inhibitor of DNA binding 2*), and *BTG2* (*B-cell translocation gene 2*) were performed using SYBR Green I, as described in a previous study^31^. The *GAPDH* transcript (encoding glyceraldehyde phosphate dehydrogenase, a housekeeping protein) was used as an internal control. The sequences of the primers for *CD52*, *ID2*, *BTG2*, and *GAPDH* used in this study are listed in Supplemental Table S1.

### Cell growth assay

Cell growth rate was determined by an MTT (3-(4,5-dimethylthiazol-2-yl)-2,5-diphenyltetrazolium bromide) assay. Briefly, parent K562-FLT3^WT/WT^ and K562-FLT3^ITD/WT^ cells (1 × 10^3^ cells/well) were seeded into 96-well plates, and the plates were incubated for the indicated intervals at 37 °C in a 5% CO_2_ environment. Subsequently, 10 μL of MTT solution (5 mg/mL) was added to each well and the plates were incubated for another 4 h. Next, cell lysis buffer was added to the wells to lyse the cells and dissolve the colored formazan crystals produced by the reduction of MTT. The optical density (595 nm) of the colored product was measured at each of the time points (0, 1, 3, 5, and 7 days) using a SpectraMAX M5 spectrophotometer (Molecular Devices, Sunnyvale, CA, USA).

### Annexin V assay

The cells were cultured in 6-well plates (5 × 10^5^ cells/ well) for 24 h, followed by incubation with fluorescein isothiocyanate (FITC)-conjugated annexin V (Biolegend, San Diego, CA, USA) and propidium iodide (PI) at approximately 25 °C for 15 min. Fluorescence intensities of FITC and PI were determined by flow cytometric analysis using a FACSCanto II instrument (BD, Franklin Lakes, NJ, USA).

### Soft agar colony formation assay

An aliquot (2 mL) of RPMI-1640 culture medium containing 0.3% agar was used as the bottom gel in each well of a 6-well plate. For each well, 5000 parent K562–FLT3^WT/WT^ or K562–FLT3^ITD/WT^ cells were suspended in 2 mL RPMI-1640 culture medium containing 0.15% agar, and the cell suspension was poured onto the bottom gel. After two weeks, colonies were stained with MTT solution. The wells were photographed under bright-field microscopy (IX-73, Olympus, Tokyo, Japan). The number of colonies was counted using Colony Counter software (BZ-X800, Keyence, Tokyo, Japan).

### Western blot analysis

Western blot analysis was performed as described in a previous study^32^. The antibodies used in this study are listed in Supplemental Table S2. Immune complexes were detected using ImmunoStar LD (Wako Pure Chemical Industries, Ltd., Osaka, Japan) in conjunction with a LAS-4000 image analyzer (GE Healthcare, Tokyo, Japan).

### ADCC assay

We analyzed ADCC induced by anti-CD52 antibody, alemtuzumab, in the parent K562–FLT3^WT/WT^, K562–FLT3^ITD/WT^, MOLT-13, and AML patient cells using flow cytometry with staining for annexin V. Natural killer (NK) cells were prepared by isolating CD56-positive cells from the peripheral blood mononuclear cells (PBMCs) of a healthy donor using anti-CD56 antibody conjugated with magnetic microbeads in combination with the autoMACS system (Miltenyi Biotec, Bergisch Gladbach, Germany). The prepared NK cells were co-cultured with the parent K562–FLT3^WT/WT^, K562–FLT3^ITD/WT^, MOLT-13, and AML patient cells in RPMI-1640 with 10% FBS in the presence of alemtuzumab; the mixtures were incubated at 37 °C for 15 h. The cells then were stained with allophycoerithrin (APC)-conjugated anti-glycophorin A antibody and phycoerythrin (PE)-conjugated anti-CD56 antibody (Biolegend) for 20 min at 4 °C. After washing twice with 500 µL annexin buffer (10 mM HEPES, 150 mM NaCl, and 2 mM CaCl_2_, [pH 7.4]), the cells were incubated with FITC-conjugated annexin V (Biolegend) for 10 min at approximately 20 °C. Then, flow cytometry was performed using a Fortessa instrument (BD Biosciences, Franklin Lakes, NJ, USA); annexin V-positive cells gated on the glycophorin A^+^ CD3^−^ population were analyzed by FlowJo software (version 10; Tree Star, Inc., Ashland, OR, USA). Cytotoxicity was calculated according to the following formula: % ADCC = 100 × (*E* − *S*) / (100 − *S*), where *E* is the concentration of annexin V-positive cells in the experimental well and *S* is the concentration of annexin V-positive cells in the absence of alemtuzumab (i.e., when target cells were incubated with NK cells alone).

### Xenograft experiment

Animal experiments were approved by the ethical committee of Aichi Medical University and performed according to their guidelines. Female Fox Chase severe combined immunodeficiency (SCID) mice (CB17/Icr-Prkdcscid/IcrIcoCrl) were purchased from CLEA Japan, Inc. (Tokyo, Japan) and bred at the Institute of Animal Experiments, Aichi Medical University, in specified-pathogen-free animal facilities. All mice used in this study were 6–8 weeks old and weighed 17–18 g each at the time of implantation. SCID mice were injected subcutaneously in the left flank with K562–FLT3^ITD/WT^ cells (1 × 10^7^ cells/mouse). Tumor growth was monitored by measuring the tumors along the perpendicular long and short axes (length and width, respectively). Tumor volumes were calculated using the formula for the volume of a modified ellipsoid (volume = 1/2 × length × width^2^). When the implanted tumors reached a mean size of 100 mm^3^, mice were randomly divided into two groups. Animals of the alemtuzumab group were administered intraperitoneally with the antibody (1 mg/kg, 2 doses/week). Animals of the control groups were administered equivalent volumes of phosphate-buffered saline (PBS; vehicle) according to the same regimen. Following the start of treatment, tumor dimensions were measured every 3 or 4 days through Day 14, at which point the animals were euthanized.

### Statistical analysis

Experimental results are expressed as mean ± standard error (SE). The statistical significance of the comparisons among groups was determined using two-tailed non-paired one-way analysis of variance (ANOVA) with post hoc Student’s *t-*test where indicated. Values of *p < 0.05, **p < 0.01, and ***p < 0.001 (indicated by asterisks) were considered statistically significant. Statistical analyses were performed using the SPSS program (version 23.0; SPSS, Inc., Chicago, IL, USA).

## Supplementary information

Primer sets used for qRT-PCR analyses.

Antibodies used for western blot and flow cytometry analyses.

Upregulated and downregulated genes in K562-FLT3ITD/WT cells compared with those in parent K562-FLT3WT/WT cells.

Generation of *FLT3*-ITD cell clones using the CRISPR-Cas9 system in K562 cells.

Genomic sequence analysis of the *FLT3* gene in parent K562 cells and *FLT3*-ITD knock-in K562 cell clones.

Gene expression changes of *ISX* and *FEZ1* in parent K562-FLT3WT/WT and K562-FLT3ITD/WT cells.

Gene expression changes of *BTG2* and *ID2* in parent K562-FLT3WT/WT, K562-FLT3ITD/WT, and rescued K562-FLT3WT/WT cells.

Status of *CD52* mRNA expression in patients with AML with or without *FLT3*-ITD, as assessed using a public database.

Cell proliferation and *CD52* mRNA expression levels in *FLT3*-ITD knock-in lymphoblastoid cell line (LCL).

Effects of alemtuzumab in MOLM-13 cells.
