# Supplementary material for: CD52 is a novel target for the treatment of FLT3-ITD-mutated myeloid leukemia
Source: Cell Death Discov. 2021 May 25;7:121. doi: 10.1038/s41420-021-00446-8 (PMC8149417; doi:10.1038/s41420-021-00446-8)
Supplement: Supplementary file 1 — Primer sets used for qRT-PCR analyses. [file 41420_2021_446_MOESM1_ESM.docx]

**Supplemental Table S1. Primer sets used for qRT-PCR analyses.**

| **Genes** | **Forward primers (5'→3')** | **Reverse primers (5'→3')** |
| --- | --- | --- |
| *CD52* | GCCACGAAGATCCTACCAAA | TGGTGTCGTTTTGTCCTGAG |
| *BTG2* | CTCCAGGAGGCACTCACAG | ATGATGGGGTCCATCTTGTG |
| *ID2* | GACAGCAAAGCACTGTGTGG | TCAGCACTTAAAAGATTCCGTG |
| *ISX* | AGCCAGGATCAACCTCCCAG | CTGCGCAGAG CAGTGGATGT |
| *FEZ1* | GCAGAGGCTC CTCGTTGATA | GAATGGCAAC TGCTCTGACA |
| *GAPDH* | GAGTCAACGGATTTGGTCGT | GACAAGCTTCCCGTTCTCAG |
