# Supplementary material for: CD52 is a novel target for the treatment of FLT3-ITD-mutated myeloid leukemia
Source: Cell Death Discov. 2021 May 25;7:121. doi: 10.1038/s41420-021-00446-8 (PMC8149417; doi:10.1038/s41420-021-00446-8)
Supplement: Supplementary file 2 — Antibodies used for western blot and flow cytometry analyses. [file 41420_2021_446_MOESM2_ESM.docx]

**Supplemental Table S2. Antibodies used for western blot and flow cytometry analyses**.

| Molecules | Product No. | Company | Species | Dilution |
| --- | --- | --- | --- | --- |
| Phospho-FLT3 (Y561) | ab171953 | Abcam | Rb | x 5000 |
| CD52 | sc-51560 | Santa Cruz | Ms | x 200 (WB) |
| CD52 | 316007 | BioLegend | Ms | x 1000 (FCM) |
| Phospho-STAT5 (Tyr694) | #4322 | CST | Rb | x 2000 |
| STAT5 | #9363 | CST | Rb | x 1000 |
| Phospho-AKT (Ser473) | #4060 | CST | Rb | x 1000 |
| AKT | #4691 | CST | Rb | x 3000 |
| GAPDH | #5714 | CST | Rb | x 2000 |
| Anti-rabbit IgG-HRP | #7074 | CST | Goat | x 4000 |

CST, Cell Signaling Technology; Rb, rabbit; Ms, mouse; WB, western blot; FCM, flow cytometry
