# Supplementary material for: CD52 is a novel target for the treatment of FLT3-ITD-mutated myeloid leukemia
Source: Cell Death Discov. 2021 May 25;7:121. doi: 10.1038/s41420-021-00446-8 (PMC8149417; doi:10.1038/s41420-021-00446-8)
Supplement: Supplementary file 3 — Upregulated and downregulated genes in K562-FLT3ITD/WT cells compared with those in parent K562-FLT3WT/WT cells. [file 41420_2021_446_MOESM3_ESM.docx]

**Supplemental Table S3. Upregulated and downregulated genes in K562-FLT3^ITD/WT^ cells compared with those in parent K562-FLT3^WT/WT^ cells.**

| Rank | Probe Name | Gene symbol | | Description | | | Fold change |
| --- | --- | --- | --- | --- | --- | --- | --- |
| Upregulated genes | | |  | |  | |  |
| 1 | A_23_P339098 | *ID2* | | Inhibitor of DNA binding 2 | | | 4.29 |
| 2 | A_33_P3220837 | *EPHB6* | | Ephrin type-B receptor 6 | | | 3.28 |
| 3 | A_24_P160969 | *CD52* | | CD52 molecule | | | 2.96 |
| 4 | A_33_P3350621 | *OSBPL6* | | Oxysterol-binding protein-related protein 6 | | | 2.88 |
| 5 | A_33_P3393010 | *ADRBK2* | | Beta-adrenergic receptor kinase 2 | | | 2.54 |
| 6 | A_23_P62752 | *SLC6A9* | | Sodium- and chloride-dependent glycine transporter 1 | | | 2.05 |
| 7 | A_23_P154605 | *BTG2* | | BTG anti-proliferation factor 2 | | | 2.02 |
|  |  |  | |  | | |  |
| Downregulated genes | | |  | |  |  |  |
| 1 | A_32_P217140 | *ISX* | | Intestine-specific homeobox | | | 0.01 |
| 2 | A_33_P3246268 | *HOXD13* | | Homeobox protein Hox-D13; HOXD13 ortholog | | | 0.01 |
| 3 | A_33_P3228315 | *CARD14* | | Caspase recruitment domain-containing  protein 14 | | | 0.01 |
| 4 | A_23_P201711 | *S100A6* | | Protein S100-A6 | | | 0.02 |
| 5 | A_23_P148541 | *CTAG1A* | | Cancer/testis antigen 1 | | | 0.02 |
| 6 | A_23_P157299 | *AEBP1* | | Adipocyte enhancer-binding protein 1 | | | 0.10 |
| 7 | A_24_P112941 | *LINGO1* | | Leucine-rich repeat and immunoglobulin-like domain-containing nogo receptor-interacting protein 1 | | | 0.11 |
| 8 | A_23_P204751 | *ASIC1* | | Acid-sensing ion channel 1 | | | 0.11 |
| 9 | A_23_P404059 | *PASD1* | | Circadian clock protein PASD1 | | | 0.12 |
| 10 | A_32_P103695 | *FAM92A1* | | Protein FAM92A1 | | | 0.12 |
| 11 | A_33_P3330911 | *BCAS1* | | Breast carcinoma-amplified sequence 1 | | | 0.12 |
| 12 | A_23_P112554 | *COL15A1* | | Collagen alpha-1(XV) chain | | | 0.17 |
| 13 | A_24_P210569 | *SCG3* | | Secretogranin-3 | | | 0.18 |
| 14 | A_23_P500433 | *CARD9* | | Caspase recruitment domain-containing protein 9 | | | 0.18 |
| 15 | A_24_P304423 | *IGF1* | | Insulin-like growth factor I | | | 0.19 |
| 16 | A_24_P309095 | *RELN* | | Reelin | | | 0.19 |
| 17 | A_32_P109683 | *PAGE2B* | | Putative G antigen family E member 3 | | | 0.20 |
| 18 | A_23_P56736 | *TUBA3D* | | Tubulin alpha-3C/D chain | | | 0.21 |
| 19 | A_23_P501007 | *EFEMP1* | | EGF-containing fibulin-like extracellular  matrix protein 1 | | | 0.21 |
| 20 | A_23_P30805 | *HIST1H4J* | | Histone H4 | | | 0.22 |
| 21 | A_23_P122852 | *SMARCD3* | | SWI/SNF-related matrix-associated actin-dependent regulator of chromatin subfamily D member 3 | | | 0.23 |
| 22 | A_33_P3249305 | *CHRD* | | Chordin | | | 0.24 |
| 23 | A_23_P73429 | *HCLS1* | | Hematopoietic lineage cell-specific protein | | | 0.25 |
| 24 | A_23_P14174 | *TNFSF13B* | | Tumor necrosis factor ligand superfamily member 13B | | | 0.26 |
| 25 | A_23_P146233 | *LPL* | | Lipoprotein lipase | | | 0.26 |
| 26 | A_23_P128598 | *TUBA3C* | | Tubulin alpha-3C/D chain | | | 0.27 |
| 27 | A_24_P259154 | *TUBA3FP* | | Tubulin alpha-3C/D chain | | | 0.28 |
| 28 | A_23_P104471 | *DUSP13* | | Dual specificity protein phosphatase 13 isoform B | | | 0.29 |
| 29 | A_23_P397293 | *LY6K* | | Lymphocyte antigen 6K | | | 0.30 |
| 30 | A_32_P171061 | *ASCL2* | | Achaete-scute homolog 2 | | | 0.30 |
| 31 | A_33_P3287472 | *PPP1R27* | | Protein phosphatase 1 regulatory subunit 27 | | | 0.31 |
| 32 | A_23_P15727 | *FKBP10* | | Peptidyl-prolyl cis-trans isomerase FKBP10 | | | 0.31 |
| 33 | A_24_P392060 | *SIMC1* | | SUMO-interacting motif-containing protein 1 | | | 0.32 |
| 34 | A_33_P3272330 | *DNMT3A* | | DNA (cytosine-5)-methyltransferase 3A | | | 0.32 |
| 35 | A_23_P112482 | *AQP3* | | Aquaporin-3 | | | 0.34 |
| 36 | A_24_P206776 | *CRYAB* | | Alpha-crystallin B chain | | | 0.36 |
| 37 | A_23_P121716 | *ANXA3* | | Annexin A3 | | | 0.36 |
| 38 | A_24_P247849 | *PBX1* | | Pre-B-cell leukemia transcription factor 1 | | | 0.37 |
| 39 | A_23_P359540 | *HIST1H4F* | | Histone H4 | | | 0.37 |
| 40 | A_33_P3409447 | *AKAP11* | | A-kinase anchor protein 11 | | | 0.37 |
| 41 | A_23_P162719 | *DIAPH3* | | Protein diaphanous homolog 3 | | | 0.38 |
| 42 | A_33_P3299319 | *RPL28* | | 60S ribosomal protein L28 | | | 0.40 |
| 43 | A_33_P3411315 | *KRTAP3-3* | | Keratin-associated protein 3-3 | | | 0.40 |
| 44 | A_23_P30813 | *HIST1H4K* | | Histone H4;HIST1H4A | | | 0.42 |
| 45 | A_33_P3321432 | *FAM198B* | | Golgi associated kinase 1B (GASK1B) | | | 0.42 |
| 46 | A_33_P3211929 | *RCOR2* | | REST corepressor 2 | | | 0.42 |
| 47 | A_23_P316850 | *ODF3L2* | | Outer dense fiber protein 3-like protein 2 | | | 0.42 |
| 48 | A_24_P314337 | *PAGE1* | | P antigen family member | | | 0.43 |
| 49 | A_33_P3314466 | *IMMT* | | MICOS complex subunit MIC60 | | | 0.43 |
| 50 | A_33_P3356216 | *ARCN1* | | Coatomer subunit delta | | | 0.43 |
| 51 | A_23_P113613 | *CDCP1* | | CUB domain-containing protein 1 | | | 0.43 |
| 52 | A_23_P111481 | *SRRT* | | Serrate RNA effector molecule homolog | | | 0.43 |
| 53 | A_32_P33083 | *VCX2* | | Variable charge X-linked protein 2 | | | 0.43 |
| 54 | A_33_P3285868 | *CYGB* | | Cytoglobin | | | 0.45 |
| 55 | A_33_P3375934 | *NAMPT* | | Nicotinamide phosphoribosyltransferase | | | 0.45 |
| 56 | A_33_P3211633 | *WDR3* | | WD repeat-containing protein 3 | | | 0.45 |
| 57 | A_33_P3315906 | *PTP4A3* | | Protein tyrosine phosphatase type IVA 3 | | | 0.45 |
| 58 | A_23_P436281 | *HIST2H4B* | | Histone H4 | | | 0.45 |
| 59 | A_23_P121926 | *SEPP1* | | Selenoprotein P | | | 0.48 |
| 60 | A_33_P3314356 | *PPM1K* | | Protein phosphatase 1K | | | 0.48 |
| 61 | A_24_P20873 | *HIST1H4I* | | Histone H4 | | | 0.48 |
| 62 | A_23_P217507 | *ZBED1* | | Zinc finger BED domain-containing protein 1 | | | 0.48 |
| 63 | A_33_P3290909 | *SMC1A* | | Structural maintenance of chromosomes protein 1A | | | 0.48 |
| 64 | A_23_P51487 | *FEZ1* | | Fasciculation and elongation protein zeta-1 | | | 0.48 |
| 65 | A_23_P207911 | *VCY* | | Testis-specific basic protein Y 1 | | | 0.50 |
