# Supplementary figures and images for: CD52 is a novel target for the treatment of FLT3-ITD-mutated myeloid leukemia
Source: Cell Death Discov. 2021 May 25;7:121. doi: 10.1038/s41420-021-00446-8 (PMC8149417; doi:10.1038/s41420-021-00446-8)

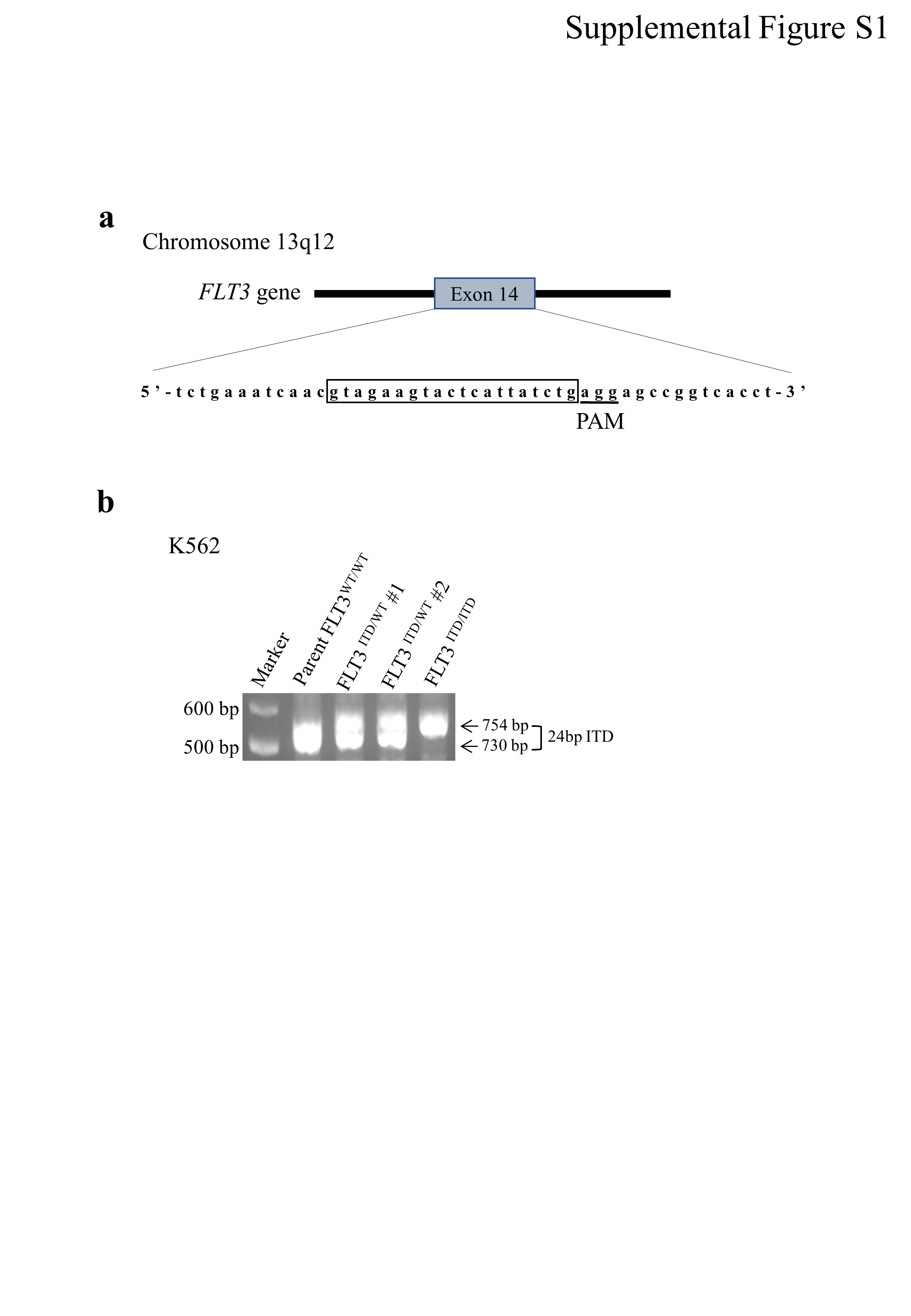

Supplement: Supplementary file 4 — Generation of FLT3-ITD cell clones using the CRISPR-Cas9 system in K562 cells. [file 41420_2021_446_MOESM4_ESM.tif]

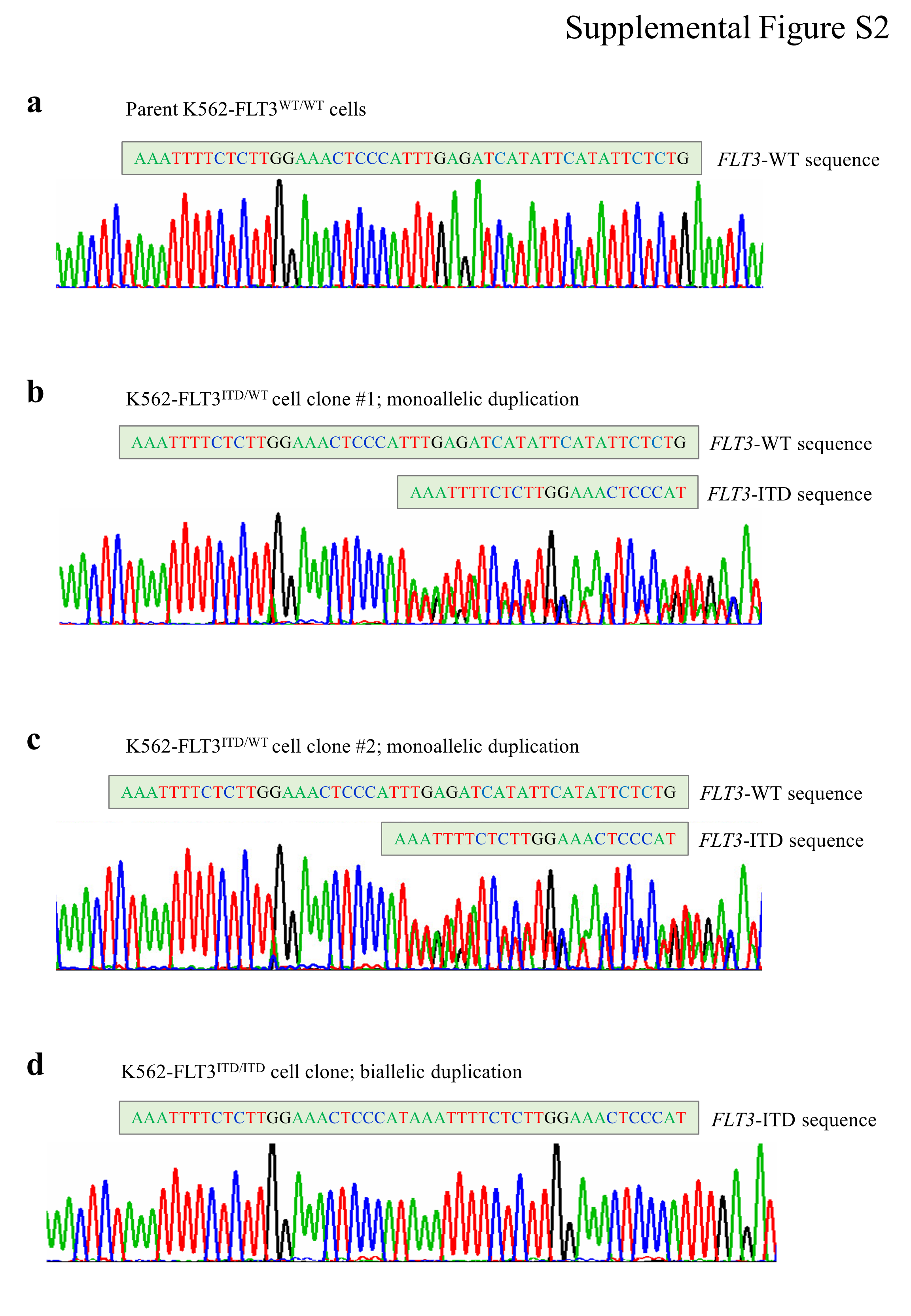

Supplement: Supplementary file 5 — Genomic sequence analysis of the FLT3 gene in parent K562 cells and FLT3-ITD knock-in K562 cell clones. [file 41420_2021_446_MOESM5_ESM.tif]

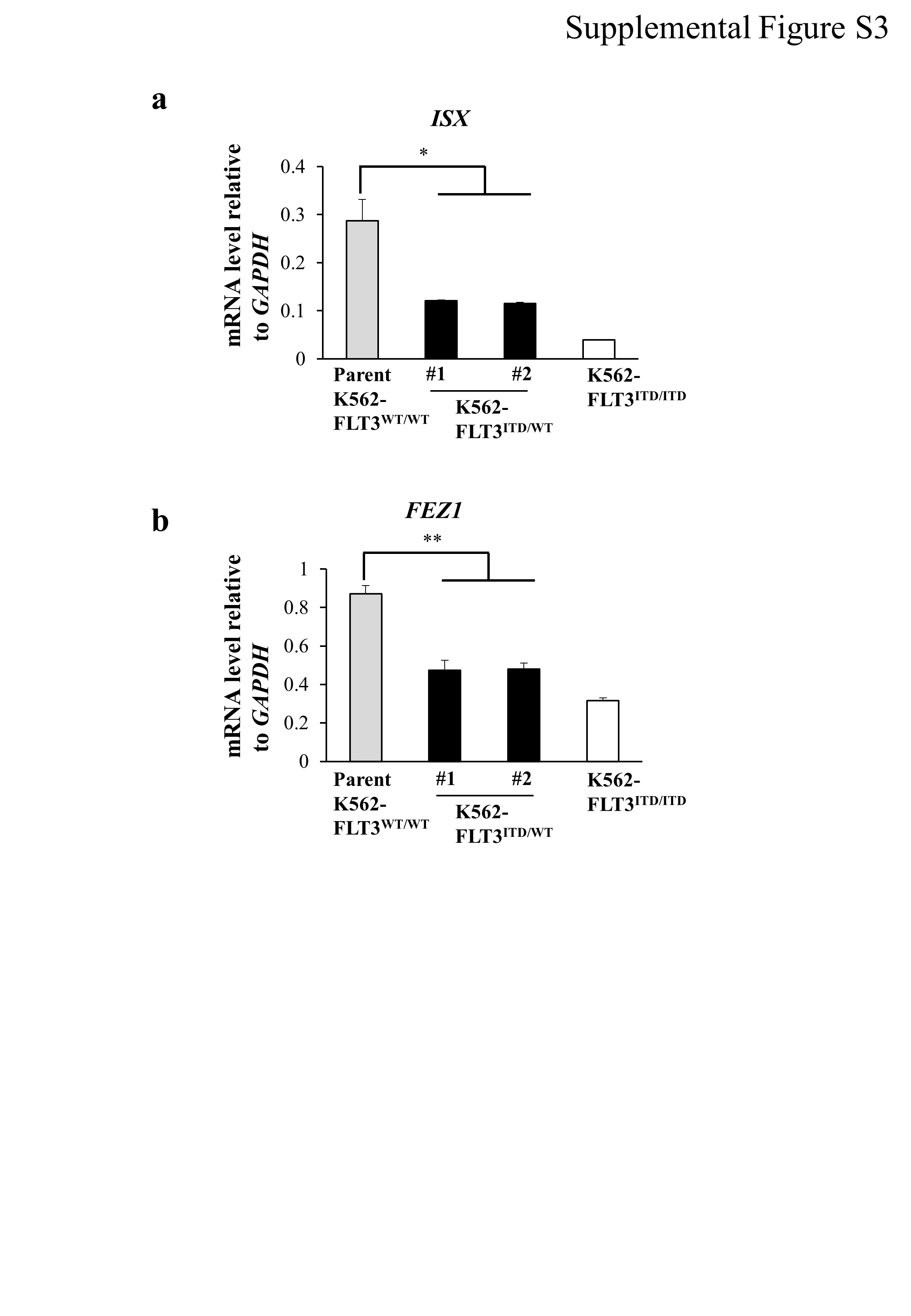

Supplement: Supplementary file 6 — Gene expression changes of ISX and FEZ1 in parent K562-FLT3WT/WT and K562-FLT3ITD/WT cells. [file 41420_2021_446_MOESM6_ESM.tif]

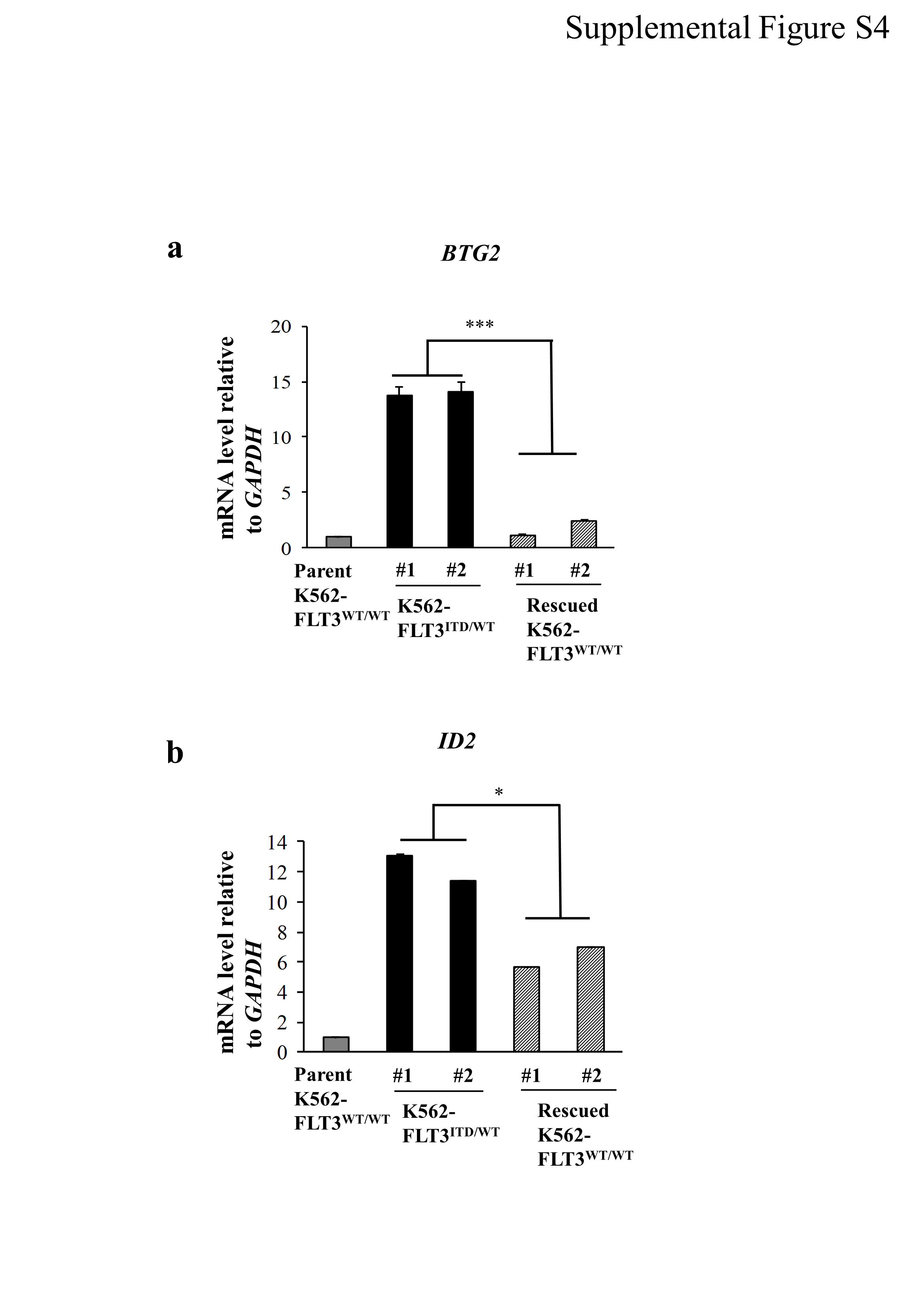

Supplement: Supplementary file 7 — Gene expression changes of BTG2 and ID2 in parent K562-FLT3WT/WT, K562-FLT3ITD/WT, and rescued K562-FLT3WT/WT cells. [file 41420_2021_446_MOESM7_ESM.tif]

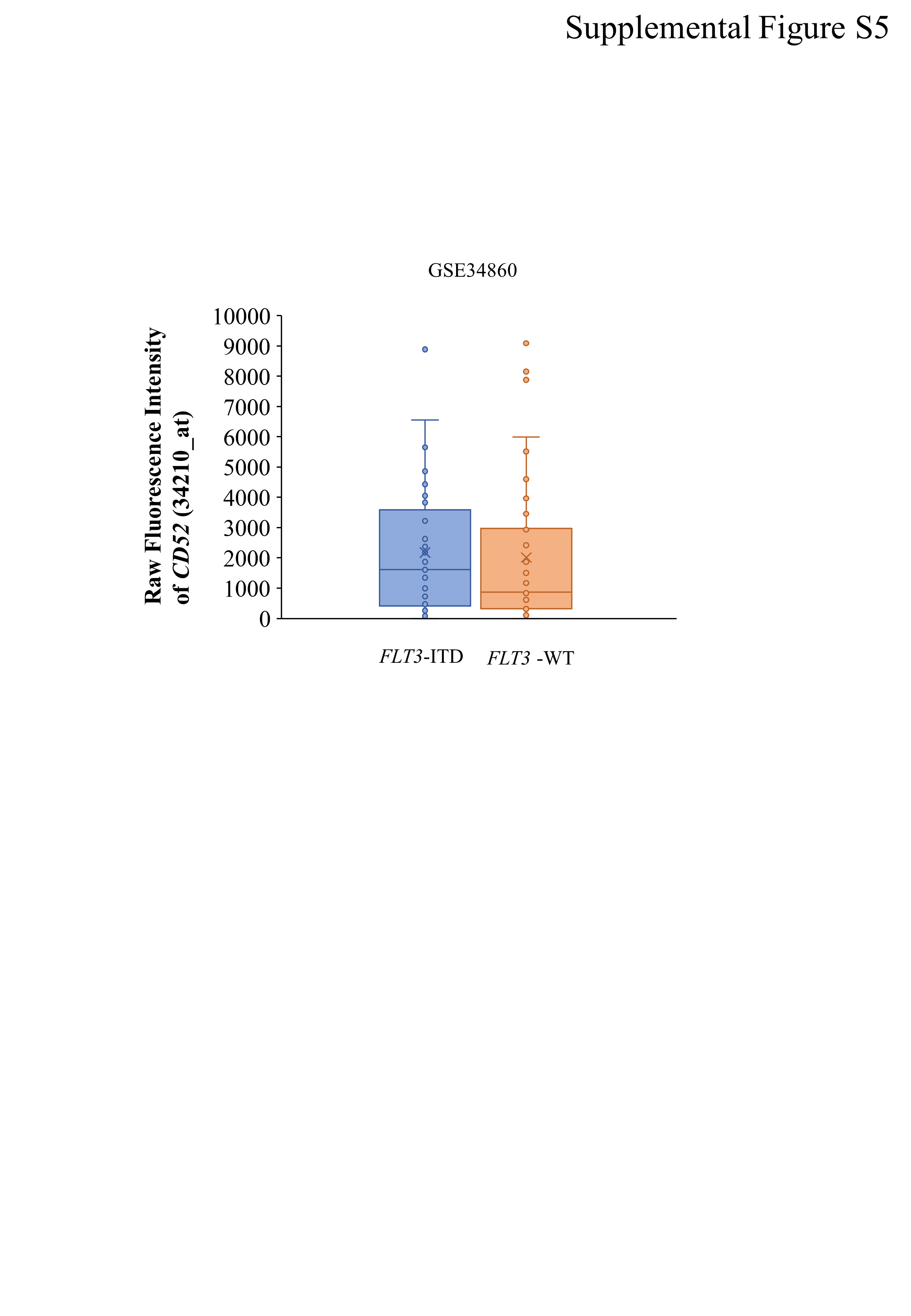

Supplement: Supplementary file 8 — Status of CD52 mRNA expression in patients with AML with or without FLT3-ITD, as assessed using a public database. [file 41420_2021_446_MOESM8_ESM.tif]

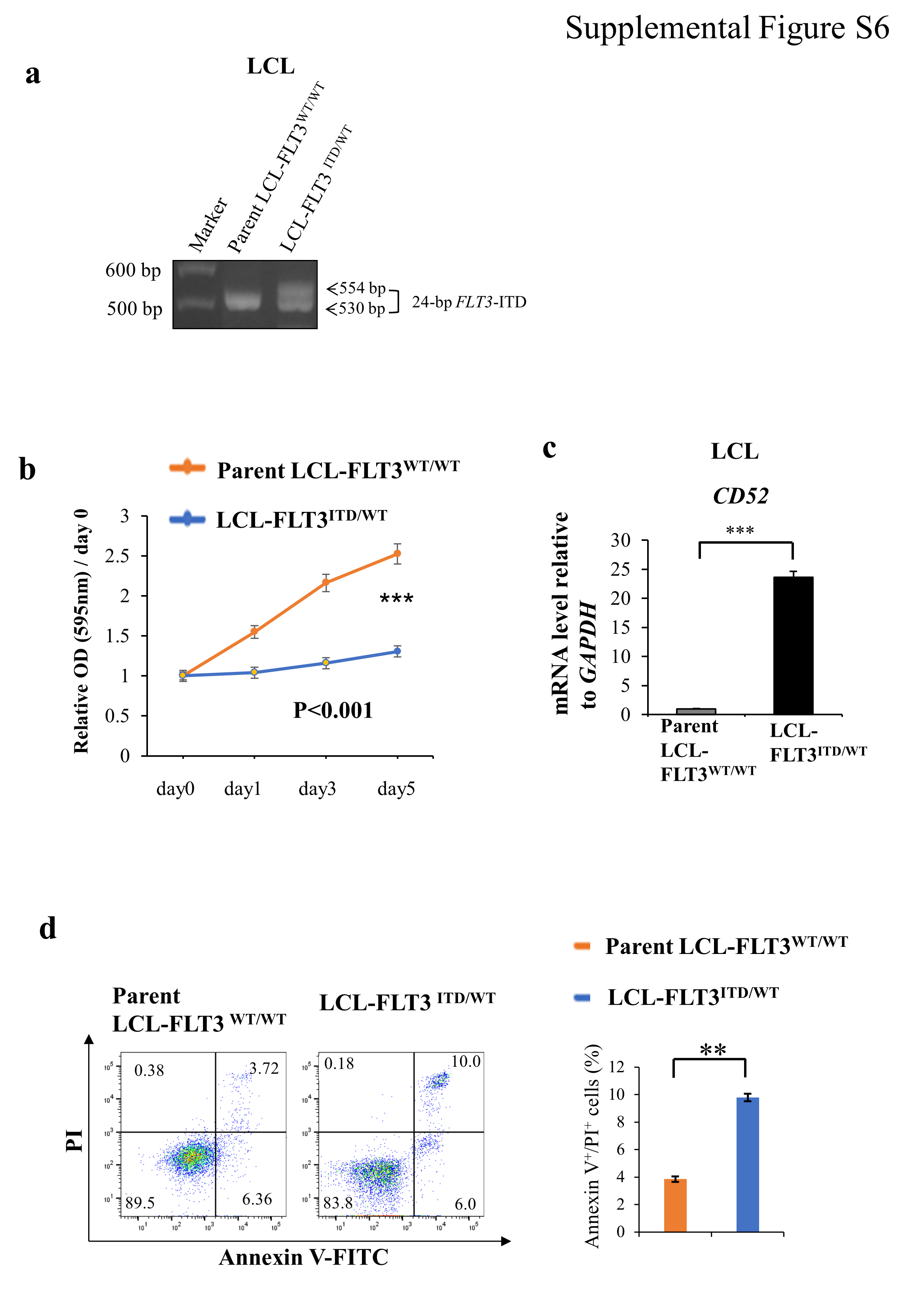

Supplement: Supplementary file 9 — Cell proliferation and CD52 mRNA expression levels in FLT3-ITD knock-in lymphoblastoid cell line (LCL). [file 41420_2021_446_MOESM9_ESM.tif]

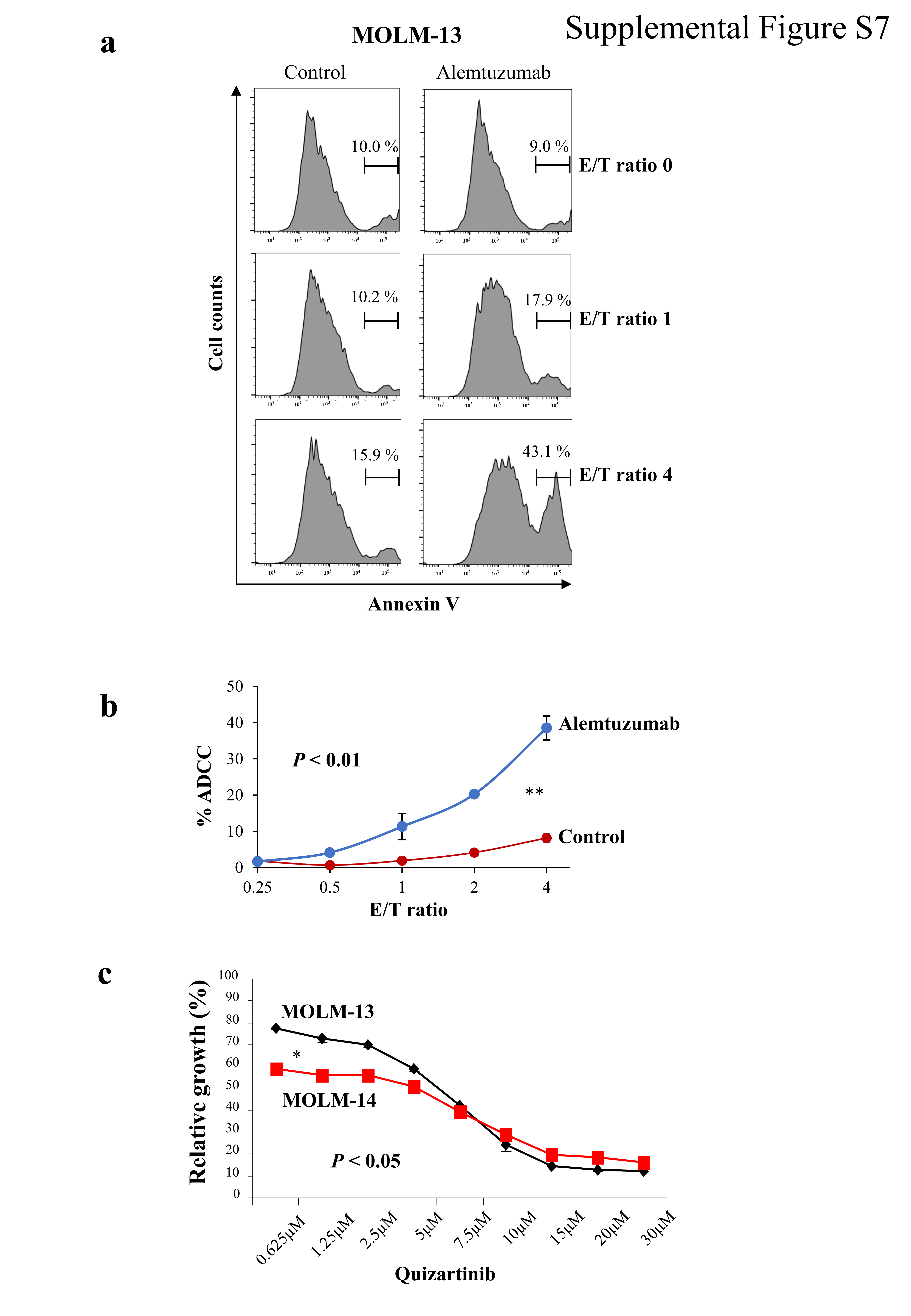

Supplement: Supplementary file 10 — Effects of alemtuzumab in MOLM-13 cells. [file 41420_2021_446_MOESM10_ESM.tif]
